# ATF6 Is a Critical Determinant of CHOP Dynamics during the Unfolded Protein Response

**DOI:** 10.1016/j.isci.2020.100860

**Published:** 2020-01-23

**Authors:** Huan Yang, Marije Niemeijer, Bob van de Water, Joost B. Beltman

**Affiliations:** 1 Division of Drug Discovery and Safety, Leiden Academic Centre for Drug Research, Leiden University, Einsteinweg 55, 2333 CC Leiden, The Netherlands

**Keywords:** Optical Imaging, Bioinformatics, Biocomputational Method, Systems Biology

## Abstract

The unfolded protein response (UPR) pathway senses unfolded proteins and regulates proteostasis and cell fate through activity of the transcription factors ATF4, ATF6, and XBP1 within a complex network of three main branches. Here, we investigated contributions of the three branches to UPR activity in single cells using microscopy-based quantification and dynamic modeling. BAC-GFP HepG2 reporter cell lines were exposed to tunicamycin, and activation of various UPR components was monitored for 24 h. We constructed a dynamic model to describe the adaptive UPR signaling network, for which incorporation of all three branches was required to match the data. Our calibrated model suggested that ATF6 shapes the early dynamics of pro-apoptotic CHOP. We confirmed this hypothesis by measurements beyond 24 h, by perturbing single siRNA knockdowns and by ATF6 measurements. Overall, our work indicates that ATF6 is an important regulator of CHOP, which in turn regulates cell fate decisions.

## Highlights

• A mathematical model of the unfolded protein response describes microscopy data
• Integration of modeling and experimental work offers insights into UPR regulation
• ATF6 shapes the early dynamics of the CHOP response

## Graphical Abstract

**Figure:**
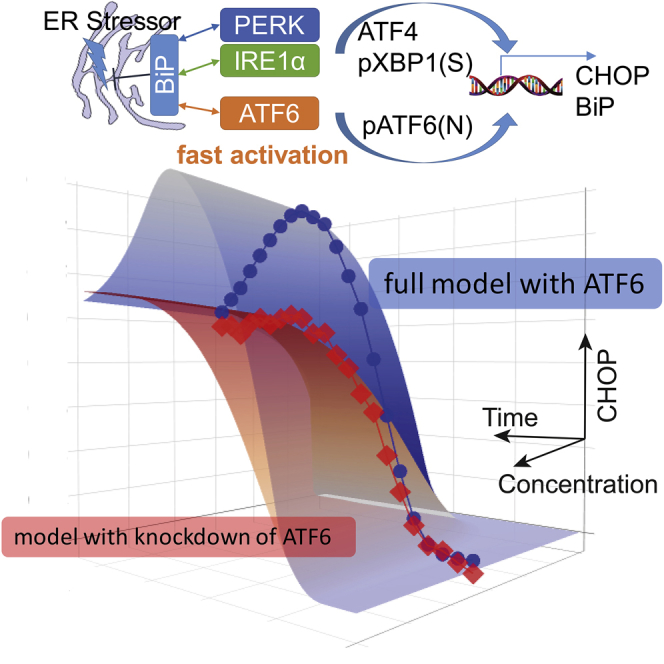

## Introduction

Cells activate adaptive stress responses to be able to cope with different types of stress. For instance, various chemicals cause the accumulation of unfolded proteins within the endoplasmic reticulum (ER). Drugs, such as nefazodone and diclofenac, lead to such ER stress, and as a consequence ER stress-related genes are upregulated, giving rise to the unfolded protein response (UPR), which counters chemical-induced protein stress (Ren et al., 2016, Fredriksson et al., 2014). Besides chemicals, also modifications in the rate of protein synthesis or in the cellular environment, such as nutrient level fluctuations or inflammation, can trigger the UPR (Wang and Kaufman, 2016). Moreover, the UPR can be exploited by malignant cells, assisting their development of drug resistance (Chevet et al., 2015).

Under homeostatic conditions, the ER is responsible for protein synthesis and tightly controls the correct folding and maturation of proteins by various chaperones (such as heat shock protein [Hsp] 70 and 90 family members, ER-localized DnaJ like proteins and calnexin), and foldases (such as protein disulfide isomerases and prolyl peptidylcistransisomerases). Afterward, proteins are transported to the Golgi through a secretory pathway (Braakman and Hebert, 2013). Upon disruption of ER homeostasis, cells react by activating the adaptive UPR. This will lead to an increase of the ER folding capacity, to temporary interruption of the translational machinery, and to degradation of unfolded proteins, altogether with the aim to recover from ER stress (Hetz and Papa, 2017, Wang and Kaufman, 2016).

**Figure 1:**
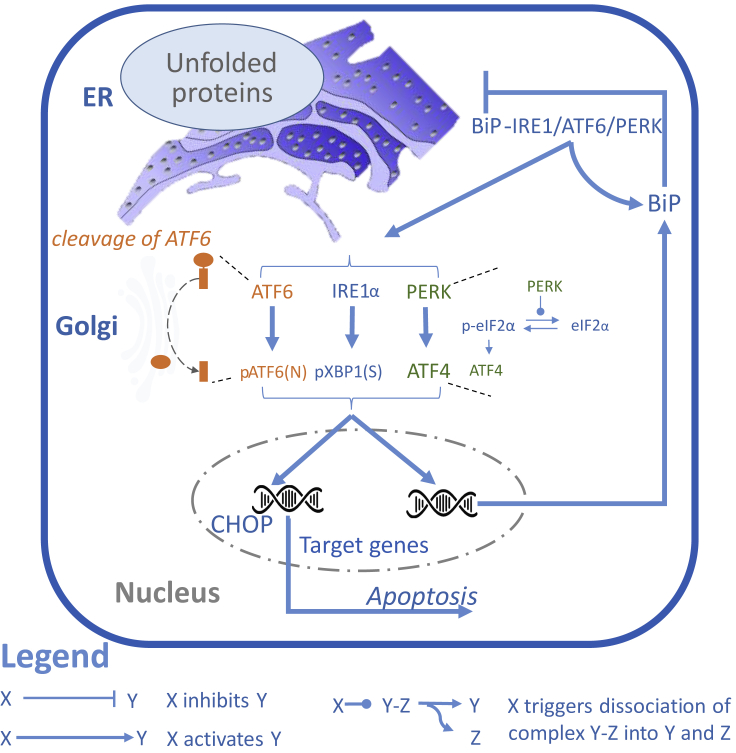
Cartoon Illustrating the UPR Pathway Involving Multiple Organelles and Three Branches, Several TFs, and Downstream Molecules Involved in Feedback Loops

The UPR is under control of three sensors, each activating distinct signaling cascades and transcription factors (TFs), namely, PKR-like ER kinase (PERK), inositol requiring 1α (IRE1α), and activating transcription factor 6 (ATF6) (Figure 1). These sensors are bound to the chaperone binding immunoglobulin protein (BiP/*HSPA5*) and are kept in an inactive state in unstressed conditions (Carrara et al., 2015, Shen et al., 2002). Upon ER stress, the sensors are released by BiP (Oikawa et al., 2009) or bound by misfolded proteins (Sundaram et al., 2018) enabling their activation. After activation of IRE1α in the first UPR branch, its endoribonuclease domain splices the b-ZIP TF *XBP1* mRNA resulting in the transcriptionally active protein pXBP1(S) (Calfon et al., 2002), which induces the expression of ER stress-related genes involved in protein folding (Lee et al., 2003), ER-associated degradation (ERAD) (Oda et al., 2006, Yoshida et al., 2003), and ER expansion (Shaffer et al., 2004). In the second branch, active PERK phosphorylates eukaryotic translation-initiation factor 2 (eIF2α) leading to attenuation of the translation of mRNAs, which reduces the protein load in the ER (Harding et al., 1999). Moreover, the expression of some genes, such as a b-ZIP TF ATF4, depends on the phosphorylation status of eIF2α (Lu et al., 2004). ATF4 induces the expression of ER stress-related genes to restore homeostasis (Ameri and Harris, 2008, Han et al., 2013) and also induces the b-ZIP TF C/EBP homologous protein (CHOP), which promotes cell death (Harding et al., 2000, Urra et al., 2013, Marciniak et al., 2004). In the third branch, ATF6 translocates to the Golgi where it is cleaved (Chen et al., 2002, Ye et al., 2000). The ensuing ATF6 fragment (pATF6(N)) translocates to the nucleus and initiates the expression of its target genes such as chaperones, genes involved in ERAD, and pXBP1(S) and also of the pro-apoptotic gene CHOP (Yoshida et al., 2000, Yoshida et al., 2001, Yamamoto et al., 2007).

As many molecules have some role in the UPR network and ample feedbacks have been identified, these interactions are expected to lead to complex dynamics. To mechanistically understand these dynamics and their role in cellular adversity, mathematical modeling is an indispensable tool to quantitatively understand this complexity (Hartung et al., 2017, Kuijper et al., 2017). Ordinary differential equation (ODE) models are well fit for this purpose because they take into account laws of biochemical reactions. Several dynamical models of the UPR have already been built by various groups. Cho et al. (2013) utilized discrete dynamical modeling to study a complex UPR network model, considering different biological processes to occur at similar time scales. With respect to ODE models applied to the UPR, several studies focused on details of UPR sub-modules, e.g., on the IRE1α branch (Pincus et al., 2010). Taking into account all three branches, Erguler et al. (2013) proposed a comprehensive UPR model and highlighted potential emerging dynamics due to feedback loops. A simpler three-branch model was derived using steady-state assumptions by Trusina et al. (2008), which was subsequently used to study repeated exposure and the effect of different types of stress during *in silico* simulations (Trusina and Tang, 2010). Interestingly, this work emphasized the potential importance of BiP accumulation during primary exposure leading to protection against renewed ER stress. Recently, Diedrichs et al. (2018) integrated gene expression data from mouse embryonic fibroblasts into a UPR model and validated their model predictions with knockout experiments, which focused on the feedback loop via CHOP-induced DNA damage-inducible protein 34 (GADD34) that leads to dephosphorylation of eIF2α and a consequent increase in protein load.

To further increase our mechanistic understanding of regulation of UPR TF activity during adaptation, we here present a new ODE model that we calibrate with a rich set of dynamic high-content imaging data. These data are generated utilizing our established liver carcinoma HepG2 BAC-GFP reporter platform (Wink et al., 2017, Wink et al., 2018, Poser et al., 2008). The usefulness of combining high-content imaging of HepG2 reporter cell lines with mathematical modeling has recently been demonstrated for the NFκB-mediated inflammatory stress pathway (Oppelt et al., 2018). Here, by applying high-content confocal imaging to HepG2 BAC-GFP UPR reporters for CHOP, ATF4, pXBP1(S), and BiP, we were able to precisely follow the activation dynamics of these UPR genes in response to a broad concentration range of tunicamycin, a highly specific ER stress inducer. By fitting our dynamic model to the data, we dissected the contribution from single branches to UPR regulation. Furthermore, model selection suggested that ATF6 has an important role in shaping the CHOP dynamics during ER stress. Consistent with this, siRNA-mediated silencing of ATF6 led to diminished CHOP induction during the acute phase, yet resulted in a prolonged induction of CHOP. This suggests that ATF6 is an important regulator for cell fate decisions under chronic ER stress.

## Results

### Image-Based Monitoring of UPR and Cellular Dynamics

To establish an ODE model that captures UPR network regulation and activation, experimental data are required that quantify the dynamics of induction of crucial UPR genes with a dense time resolution. We achieved such a resolution by combining our previously established liver carcinoma HepG2 BAC-GFP UPR reporters (Wink et al., 2017, Wink et al., 2018) with high-content confocal microscopy. We used the compound tunicamycin as an ER stress inducer, which inhibits N-glycosylation and therefore leads to the accumulation of unfolded glycoproteins (Yoo et al., 2018). Tunicamycin specifically induces ER stress and is therefore an excellent compound to create a UPR-specific ODE model.

**Figure 2:**
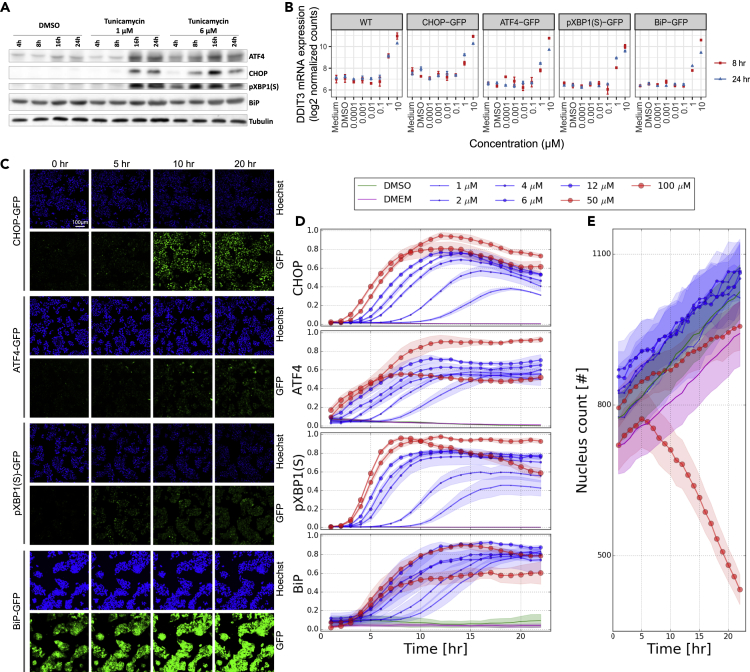
Dynamic Measurements of Various UPR Components to Integrate with Modeling (A) Western blot of CHOP, ATF4, pXBP1(S), and BiP protein at 4, 8, 16, and 24 h upon exposure to DMSO or tunicamycin (1 and 6 μM) in WT HepG2 cells. Tubulin was used as protein loading control. (B) Log2 normalized counts of DDIT3 mRNA expression analyzed using TempO-seq transcriptomics at 8 or 24 h after exposure with various concentrations of tunicamycin in HepG2 WT and UPR BAC-GFP reporter cell lines. (C) Representative images of HepG2 UPR BAC-GFP reporter cell lines (CHOP, ATF4, pXBP1(S), and BiP) stained with Hoechst for nuclei visualization. Images were obtained using confocal microscopy with a 20× objective at the indicated time points after exposure to tunicamycin at 6 μM. Hoechst is represented in blue (upper rows) and GFP in green (lower rows). (D and E) Quantification of single-cell-based GFP intensity of the HepG2 UPR BAC-GFP reporter cell lines after min-max normalization (D) and cell counts (E) after exposure to DMEM/DMSO or to a broad concentration range of tunicamycin and imaged live every hour for 24 h after exposure using confocal microscopy. BiP-GFP intensity was quantified in the cytoplasm; all other reporters were quantified in the nuclei. Data in (B), (D), and (E) represent mean and standard error of the mean (SE) of three biological replicates.

We first examined whether our HepG2 UPR reporters for CHOP, ATF4, pXBP1(S), and BiP are representative for the behavior of wild-type (WT) HepG2 cells. To this purpose, we established the protein expression of endogenous CHOP, ATF4, pXBP1(S), and BiP using western blotting in HepG2 WT cells after tunicamycin exposure for 4, 8, 16, and 24 h. Both treatment with 1 and 6 μM tunicamycin resulted in a clear induction of UPR proteins (Figure 2A). However, BiP was already highly expressed at basal levels and therefore it was unclear whether further induction occurred. A high tunicamycin concentration of 6 μM led to an earlier induction of UPR proteins than a low concentration of 1 μM (Figure 2A).

Next, we assessed if all four HepG2 UPR reporters behaved similarly upon tunicamycin exposure as WT cells. Applying a TempO-seq targeted transcriptomics approach to all five HepG2 (WT and reporter) cell lines exposed to a broad concentration range of tunicamycin for 8 or 24 h revealed that *DDIT3* (i.e., the gene coding for the CHOP protein) expression across HepG2 wild-type and BAC-GFP cell lines followed a similar dose response at both time points (Figure 2B). For other UPR-related genes, the different cell lines also have a similar dose response behavior and are highly correlated in gene expression (Figure S1). As expected based on having at least one additional copy of the gene, HepG2 CHOP-GFP exhibited a slightly higher *DDIT3* expression at baseline compared with the other lines, but this did not influence the dose response of *DDIT3* itself (Figure 2B) or the expression of other UPR-related genes (Figure S1). Thus, all HepG2 UPR reporter behave similarly with respect to UPR gene expression.

To generate dynamic protein expression data to which results from an ODE model can be compared, we exposed HepG2 BAC-GFP UPR reporters for CHOP, ATF4, pXBP1(S), and BiP to a concentration range from 1 to 100 μM of tunicamycin and subsequently applied live imaging with confocal microscopy to capture the GFP induction in single cells and total cell count every hour until 24 h of exposure (Figures 2C and 2D). The dynamic pattern of CHOP-GFP expression exhibited a peak around 10–20 h (Figures 2C and 2D), which was consistent with the CHOP expression in WT HepG2 cells observed with western blotting (Figure 2A). Increasing concentrations of tunicamycin led to earlier maxima of CHOP expression levels (Figure 2C). For all four reporters, a concentration-dependent increase in maximal GFP intensity occurred. However, at the highest concentration (100 μM) of tunicamycin, the maximal GFP intensity was equal or lower compared with that of 50 μM, which is indicative of cellular toxicity. Consistent with this interpretation, the total number of cells dramatically decreased at 100 μM of tunicamycin (Figure 2E). At 50 μM of tunicamycin, there was also a slower increase in cell count over time compared with lower concentrations. Therefore, only concentrations below 50 μM of tunicamycin were taken along for the ODE-model development since we here focus on the adaptive UPR signaling network. In summary, the gene expression as well as protein expression levels of BAC-GFP HepG2 UPR reporter cell lines and WT HepG2 cells exhibited similar baseline levels and dynamic patterns upon exposure to tunicamycin. Therefore, we concluded that the BAC-GFP UPR cell lines were sufficiently representative for WT HepG2 cells to be used for subsequent dynamical modeling.

### UPR Model with ATF6 Provides Excellent Fit to the Data

**Figure 3:**
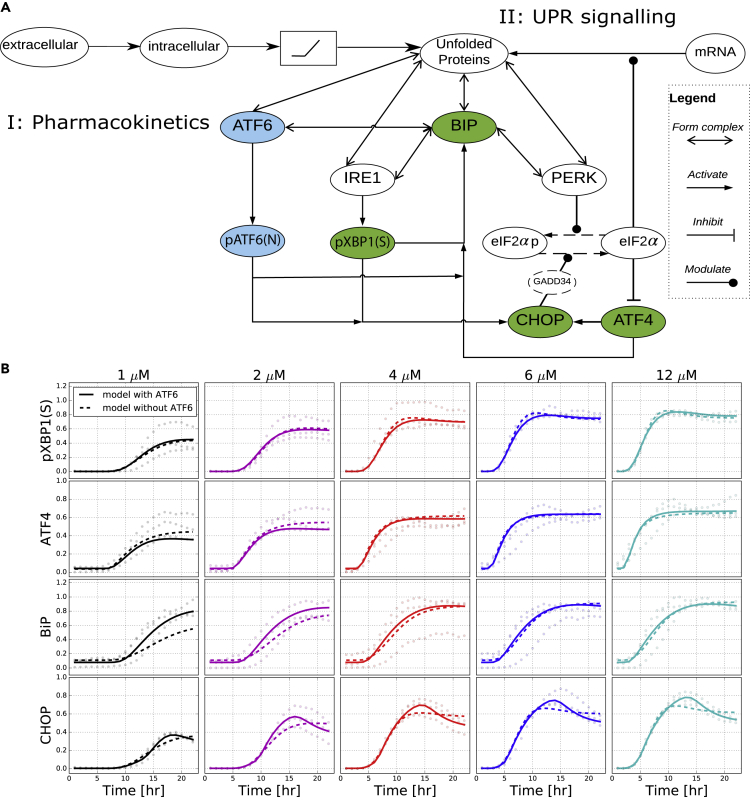
Model Structure and Fit (A) Schematic diagram of the modeled UPR pathway with both pharmacokinetics and signaling network. (B) Model fits to the experimentally observed levels of pXBP1(S), ATF4, BiP, and CHOP upon tunicamycin exposure at five concentrations. Dots present values for three replicates. Optimized fits from models with ATF6 branch (solid curves) or without (dashed curves) are plotted.

Because we had dynamic information on four BAC-GFP reporter cell lines, we initially constructed an ODE model with four variables representing the protein expression level for these reporters as well as a variable for the amount of unfolded proteins in the cell (Figure 3A). This model was a modification of an earlier published model by Trusina et al. (2008). We did not incorporate ATF6 explicitly, but it was considered to behave similarly to IRE1α, i.e., these sensors were considered to be in quasi steady state (Trusina et al., 2008). In addition, we modeled the downstream molecules ATF4 and CHOP. Finally, because the experimentally observed dynamics of intensity of all UPR reporters exhibited a concentration-dependent delay of activation for tunicamycin concentrations below 12 μM (Figure 2D), we incorporated this phenomenon in a pharmacokinetic module preceding the signaling module. Specifically, we added a threshold in the effective intra-cellular concentration of tunicamycin, i.e., we consider the UPR signaling to be triggered only when a particular intra-cellular stress level is crossed, which leads to some delay of pathway activation (see simulated pharmacokinetic profiles in Figure S2).

This initial model could roughly describe the reporter dynamics, yet this could not capture the consistently observed dynamic peak in CHOP expression (Figure 3B, dashed line). Therefore, we also created a model variant including the ATF6 branch explicitly (for which no BAC-GFP reporter cell line was available). The model with all three UPR branches contains 47 parameters, whereas the model without ATF6 has 39 parameters (for equations see Transparent Methods). After fitting of both models to the experimental data (for parameter estimates see Table S1, for their estimated standard errors see Figure S3, and for their sensitivity see Figure S4), visual comparison of the two model variants showed that only the model with ATF6 was able to describe the CHOP peak (Figure 3B, solid line). This visual impression was confirmed by application of a likelihood-ratio-based approach to compare the models with the data (Δ*G* = 119 and 271 for the full and ATF6-free models, respectively; p < 0.001), and by calculation of the information criteria AIC and BIC (Pawitan, 2001) for the two competitive models (AIC full model: 2×119 + 2×47 = 332; AIC ATF6-free model: 2×271 + 2×39 = 620; BIC full model: 2×119 + ln(440) 47 = 524.08; BIC ATF6-free model: 2×271 + ln(440) 39 = 779.38). The above results thus suggest that the ATF6 branch plays an important role in shaping the early CHOP dynamics, and we continued with the calibrated model including ATF6 for further exploration and validation.

### Model Correctly Predicts CHOP Dynamics beyond 24 Hours

**Figure 4:**
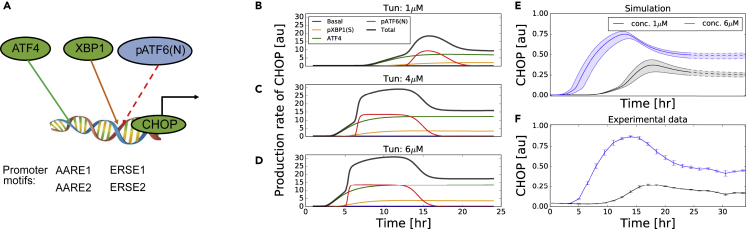
Model-Based Prediction of CHOP Transcription (A) Illustration of the TFs contributing to CHOP transcription. (B–D) Simulations of the contributions of pXBP1(S), ATF4, and pATF6(N) to the CHOP production rate after exposure to tunicamycin concentrations of 1 (B), 4 (C) and 6μM (D), respectively. (E) Model prediction of CHOP levels within the first 24 h (solid line) and between 24 and 34 h (dashed line). Simulations were conducted with various strengths of exposure (between 30% and 130% of the reference value) shown as shaded areas. (F) Image-based experimental observation of CHOP for 34 h represented as the mean ± SE of three biological replicates.

The transcription of CHOP can be induced by binding of UPR TFs, i.e., ATF4, pXBP1(S), and pATF6(N), at the AARE and ERSE promoter motifs (Figure 4A [Takayanagi et al., 2013, Oyadomari and Mori, 2004]). However, previous work has suggested that induction of CHOP is predominantly regulated by ATF4 and pATF6(N), and to a minimal extent by pXBP1(S) (Diedrichs et al., 2018, Wu et al., 2007, Ma et al., 2002). Having the parameterized full UPR model in place allowed us to explore both the speed of activation of the three sensors and the contribution of each of the three downstream TFs to CHOP induction at different time points. With respect to the speed of activation of the sensors, ATF6 is the sensor responding most quickly, followed by IRE1α and finally PERK (Figure S5). With respect to the contribution of the downstream TFs to CHOP transcription, we investigated this by separating the mathematical term representing the CHOP production rate into the individual TF contributions forming this term. This analysis showed that the ATF6 branch shapes the early dynamics of CHOP production, whereas ATF4 dominates the CHOP production at late time points (Figures 4B–4D). This explains why ATF4 is typically considered the primary TF responsible for CHOP production (Scheuner et al., 2001, Harding et al., 2000), yet our analysis suggests that pATF6(N) also has an important contribution to CHOP production at early time points. This happens because pATF6(N)-mediated CHOP transcription starts and ends relatively abruptly owing to the high cooperativity (*n* = 46.32 in the best fit) in the Hill function describing pATF6(N) activity. Once pATF6(N) drops below the Hill threshold *K*_*A*2*C*_ (which equals 0.717 in the best fit), the effect of the still relatively high pATF6(N) levels on CHOP transcription quickly becomes negligible. Note that such a high cooperativity is required to explain the exact height of the CHOP peak (Figures S6–S8). Furthermore, our analysis confirmed the minimal role of pXBP1(S) in CHOP transcription, which is due to low pXBP1(s) levels rather than to low TF activity of the present pXBP1(s) (Figure S9).

Given the model prediction that the ATF4-driven CHOP production rate remains relatively high around 24 h, we simulated the model for a duration longer than the 24 h on which the parameterization was based. Beyond 24 h the CHOP level was predicted to stay around the same level for tunicamycin concentrations of 1 and 6 μM (Figure 4E) rather than quickly returning to baseline level. Our simulations predicted that this was due to a gradual increase of the intra-cellular stress levels, which saturated after ∼20 h and did not yet decrease (Figure S2). The sustained high ATF4 level is attributed to its upstream molecules PERK and eIF2α that tightly follow the dynamics of the intra-cellular stressor and of unfolded protein (Figure S9). To validate this model prediction, we performed imaging experiments of a duration beyond 24 h, which showed that indeed CHOP-GFP levels in HepG2 cells remained at a relatively high level up to 34 h (Figure 4F). Thus, although the model was based on 24-h measurements, it correctly predicted sustained CHOP levels beyond 24 h.

### Knockdown Experiments Confirm Role of ATF6 in CHOP Dynamics

**Figure 5:**
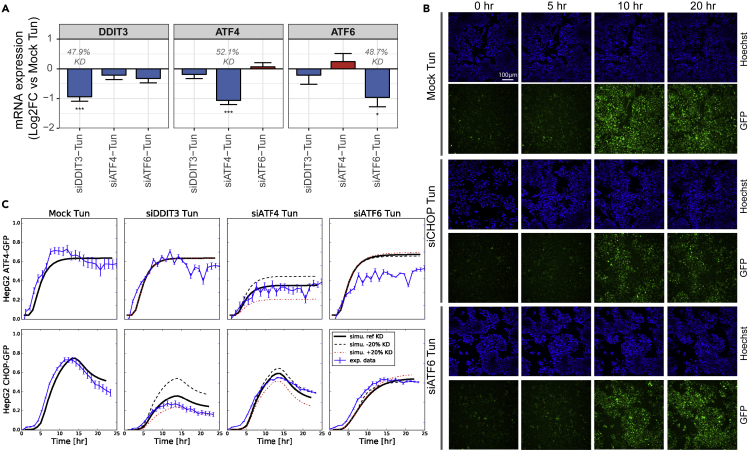
Perturbation of UPR with siRNA Knockdowns Are Consistent with Model Predictions (A) Log2 fold changes of mRNA expression of different siRNA-mediated gene knockdowns relative to siRNA mock negative control in HepG2 WT cells exposed to 6 μM of tunicamycin for 16 h, determined using TempO-seq transcriptomics. Knockdown efficiencies of siRNAs are depicted in gray numbers. Data represent the mean ± SE of three biological replicates. (B) Representative confocal microscopy images obtained with 20× objective of HepG2 CHOP-GFP reporter cells exposed to 6 μM of tunicamycin for 16 h after CHOP, ATF6, or Mock siRNA. To visualize the nuclei, cells were stained with Hoechst (upper rows), and CHOP-GFP is represented in green (lower rows). (C) Model simulation of ATF4 and CHOP (black curves) compared with quantified GFP data after exposure to 6 μM of tunicamycin for different siRNA-mediated knockdown conditions (blue line and error bars representing mean ± SE of three biological replicates). Simulations with varied knockdown efficiency (black dashed: 20% less, red dashed: 20% more) are also plotted.

We next challenged our model further by evaluating the effect of perturbing single UPR-related genes, including ATF6, on activation of other UPR components using siRNA-mediated silencing. To confirm success of knockdown by siRNA and to quantify its efficiency, we first measured the expression of *DDIT3*, *ATF4*, and *ATF6* after knockdown of these separate genes for 3 days and subsequent exposure to 6 μM tunicamycin for 16 h. TempO-seq transcriptomics experiments showed that expression of these genes was indeed significantly decreased by siRNA-mediated silencing upon exposure to tunicamycin (Figure 5A). To study the effect of perturbation of UPR-related genes on CHOP and ATF4 induction dynamics during ER stress, we then measured CHOP-GFP and ATF4-GFP in HepG2 BAC reporters using confocal imaging for 24 h after 6 μM tunicamycin exposure when no gene (Mock), *DDIT3*, *ATF4*, or *ATF6* was silenced using siRNA (Figure 5B and blue lines in Figure 5C). Knockdown of *DDIT3* and *ATF4* led to reduced levels of, respectively, CHOP-GFP and ATF4-GFP, confirming the success of the knockdowns also at protein level. We then compared the experimental measurements upon knockdown with model predictions incorporating the knockdown efficiencies that we measured for the different genes (Figure 5C).

*ATF4* and *ATF6* knockdown both affected the CHOP-GFP dynamics, yet its effect was qualitatively different. *ATF4* knockdown led to a decrease in CHOP induction, yet a clear peak remained present in the CHOP dynamics around 16 h post tunicamycin exposure, indicating that ATF4 is not responsible for that peak (Figure 5C). Similarly, *ATF6* knockdown led to a reduced CHOP induction specifically in the initial phase. However, after 16 h of exposure, CHOP levels did not decline again and CHOP levels at 24 h were slightly higher when ATF6 was silenced than for the Mock control. Our model offers an explanation for these observations: First, the lowered activity of pATF6(N) due to ATF6 knockdown implies that the CHOP transcription rate contributed by pATF6(N) does not exceed the required threshold and that CHOP transcription fully depends on XBP1(S) and ATF4 activity. Second, the reduced pATF6(N) upon knockdown also lowers BiP expression, thus leading to an increased amount of unfolded proteins, XBP1(S) and ATF4, which in turn slightly increases CHOP expression around 24 h compared with a setting without knockdown (Figure S10). Thus, ATF6 affects the CHOP dynamics especially in the initial phase and also slightly in the later phase as was predicted by our model. Altogether, the experimentally observed alterations in ATF4 and CHOP induction could be accurately predicted with our model and this analysis confirmed the model prediction that ATF6 shapes CHOP dynamics. As ATF6 shapes the dynamic pattern of pro-apoptotic CHOP, i.e., initially increases CHOP but later decreases it owing to initial BiP-mediated folding of unfolded proteins, we speculate that early ATF6 activity may in fact protect cells under chronic ER stress. This is consistent with experimental findings in ATF6 knockout mice in which cell death increased upon exposure to tunicamycin after 18 h (Wu et al., 2007).

### ATF6 Activation Peaks Early as Predicted by Modeling

**Figure 6:**
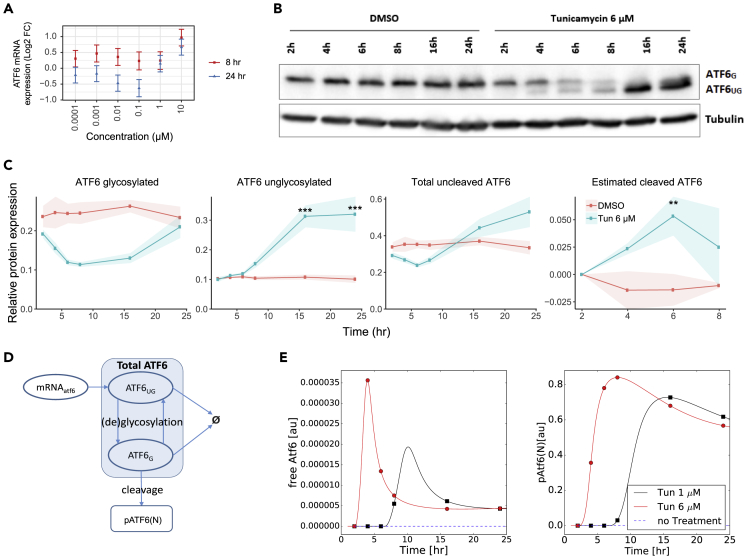
Matching ATF6 Dynamics in Experiment and Model (A) ATF6 mRNA expression after 8 or 24 h of exposure to a broad concentration range of tunicamycin in HepG2 WT cells using TempO-seq, represented as the mean of log2FC ± SE of three biological replicates. (B) Western blot of uncleaved ATF6 (G, glycosylated, UG, unglycosylated) measured in HepG2 WT cells at 2, 4, 6, 8, 16, or 24 h after exposure to tunicamycin (6 μM) or DMSO. As protein loading control, tubulin protein expression was assessed. (C) Quantified protein expression of the indicated ATF6 forms from three biological replicates after protein loading correction using tubulin (symbols and shaded area represents mean ± SE with the significance levels represented as **p_*adj*_ < 0.05, ***p_*adj*_ < 0.01). Cleaved ATF6 was estimated based on the difference between total uncleaved ATF6 at 4, 6, and 8 h versus the 2-h time point. (D) Diagram of relation between different ATF6 forms during tunicamycin treatment, where ATF6_G_ and ATF6_UG_ represent glycosylated and unglycosylated forms, respectively. (E) Model-predicted dynamics of free ATF6 and the downstream pATF6(N) upon exposure of tunicamycin at 1 and 6 μM.

Since our model predicts that the peak in CHOP dynamics that follows tunicamycin exposure is due to early ATF6 activity, we evaluated the mRNA expression and activation dynamics of endogenous ATF6 in HepG2 WT cells by TempO-seq transcriptomics and western blot. ATF6 mRNA expression increased by 2-fold at 10 μM of tunicamycin at 8 and 24 h, but not at 1 μM (Figure 6A), suggesting minor upregulation of ATF6 only at high concentrations. At protein level, exposure to 6 μM of tunicamycin clearly led to the expected inhibition of N-glycosylation, which became visible by the appearance of a low western blot band representing unglycosylated, uncleaved ATF6 (ATF6_UG_) and a decrease of the high band representing glycosylated ATF6 (ATF6_G_) starting from 4 h of exposure (Figure 6B; quantification in Figure 6C, first two panels). Exposure to a lower concentration of 1 μM tunicamycin also increased the formation of ATF6_UG_, yet it was only apparent at late time points (Figure S11).

The relation between ATF6_G_ and ATF6_UG_, which changes during tunicamycin exposure, is illustrated in Figure 6D, i.e., both forms can degrade, but only ATF6_G_ can lead to pATF6(N). The amount of total uncleaved ATF6 (i.e., ATF6_UG_ + ATF6_G_) decreased at early time points (6 h, p = 0.016; 8 h, p = 0.070) compared with DMSO control but restored later on (Figure 6C third panel). Since levels of endogenous cleaved ATF6 in HepG2 cells were difficult to capture using western blot, we assessed ATF6 cleavage from the difference in total uncleaved ATF6 levels. Considering the ATF6 production and degradation rates to remain roughly unchanged at early time points in tunicamycin and DMSO conditions, the decreased amount of total uncleaved ATF6 at those time points can be attributed to ATF6 cleavage. Therefore, we used the difference in total uncleaved ATF6 between the first measured time point and subsequent time points as a measure for ATF6 cleavage (Figure 6C, panel 4). The level of pATF6(N), as estimated through this approach, peaked at 6 h post tunicamycin exposure (p = 0.044), which is consistent with the dynamics of predicted free ATF6 and pATF6(N) in our computational model (Figure 6E).

In conclusion, the activation dynamics of ATF6 were early and concentration dependent as predicted by our model. Together, our combination of experimental and computational modeling work shows that ATF6 is activated early after tunicamycin exposure and that this causes an early rise in CHOP expression. The CHOP expression subsequently drops to a lower level yet remains relatively high owing to ongoing presence of stress, keeping ATF4 expression at elevated levels.

## Discussion

The basis of our work consisted of dynamic measurements detailing the induction of UPR regulators in HepG2 reporter cell lines during tunicamycin-induced ER stress. We exploited these data to establish a computational model representing the essential mechanisms shaping the UPR and fitted the model using 24-h reporter dynamics. The strength of our approach was that we exploited a large amount of high-content imaging data to obtain a quantitative understanding of UPR regulation. This combination of modeling and experiments helped to unravel the role of different molecules in the UPR dynamics. Specifically, the model predicted that the ATF6 branch was required to explain the observed UPR dynamics and this prediction was verified by knockdown experiments, prolonged experimental time courses, and additional western blot measurements.

Some of the previously published UPR modeling work focused on theoretical understanding of network dynamics in different scenarios (Trusina et al., 2008, Erguler et al., 2013). Specifically, in the extensive model of Erguler et al. (2013), it was shown that the network could exhibit different kinds of structural behavior depending on the parameter settings. For example, for some parameter conditions oscillations occur, showing that the network is in principle capable of generating such behavior. However, our combined modeling and experimental analysis demonstrates that at least for HepG2 cells exposed to tunicamycin such oscillations do not occur. Owing to the complexity of the model by Erguler et al. (2013) precluding calibration to a dataset that was limited in terms of number of monitored variables, we instead chose to extend the model by Trusina et al. (2008) with CHOP and ATF6, rendering a new model with similar UPR TF activity that could be calibrated to our imaging data. A combination of experimental and computational work similar to ours has been recently reported by Diedrichs et al. (2018), where model predictions were based on qPCR and western blot experiments. Key differences with our approach include the choice of test compound and the balance of model complexity and measurements. With respect to the employed compounds, Diedrichs et al. (2018) exposed MEFs to thapsigargin, a SERCA inhibitor disturbing calcium homeostasis, whereas we used tunicamycin, which inhibits N-glycosylation within the ER. The downside of using exposure to thapsigargin is that it not only leads to a strong UPR induction but also induces oxidative stress, at least in HepG2 cells (Wink et al., 2017). With respect to model complexity and the amount of experimental data, time-lapse imaging data has a major advantage that it easily delivers many data points at the single-cell level within specific sub-cellular compartments, i.e., we have more than 400 datapoints measured from four BAC-GFP reporters at five concentrations and at more than 20 time points.

Besides capturing the dynamics of UPR-related molecules, our quantitative modeling approach suggests that ATF6 is responsible for the early peak of CHOP. Both our knockdown experiments and ATF6 measurements using western blotting at different time points are consistent with this hypothesis. Specifically, the decrease in total uncleaved ATF6 strongly suggested that cleavage of ATF6 peaked at early time points (around 6 h). These findings are also consistent with those of Yoshida et al. (2001), who reported a similar pattern with an overshoot in the nuclear active ATF6 fragment after tunicamycin treatment in HeLa cells. To verify the observed activation dynamics of ATF6 and to capture high-resolution activation dynamics at sub-cellular localization, future imaging-based dynamic readout of ATF6 and its fragments would be highly valuable. Based on such data, the part of our model describing ATF6 could also be extended and better parameterized.

The parameters in our mechanistic model have a biological interpretation, and their estimates thus provide quantitative insight into UPR regulation. First, the degradation rate of the protein CHOP (*r*_*C*_) was estimated to be 5-fold larger than that of BiP (*r*_*B*_), i.e., a similar difference as found by Rutkowski et al. (2006). Given the protective role of BiP through protein folding and the pro-apoptotic role of CHOP, this suggests that the distinct degradation rates represents one mechanism that explains initial adaptation to ER stress, followed by a switch toward adversity during prolonged ER stress. Second, the parameters *K*_*BP*_, *K*_*BI*_, and *K*_*BA*_ shape the response sensitivity among the three UPR branches PERK, IRE1α, and ATF6, with the latter being the quickest (Figure S5). Interestingly, we showed that ATF6(N) transcriptional activity with respect to CHOP is also switched off early and abruptly owing to the high predicted cooperativity of this response (Figure 4).

In response to ER stress, cells have several coping strategies to eliminate the accumulation of misfolded proteins by activating the three UPR branches. However, in case ER stress becomes too severe or chronic, apoptotic signaling pathways will be activated and cells will switch from adaptive to pro-apoptotic signaling. In this switch, CHOP plays an important role through various mechanisms (Urra et al., 2013, Uzi et al., 2013, Ji et al., 2005) and therefore regulators of CHOP can affect the sensitivity of cells to ER stress. Here, we found that ATF6 has such a crucial role in the dynamics of CHOP induction, where perturbation of ATF6 led to absence of the initial CHOP peak yet led to slightly increased CHOP levels at a later stage. Our findings are consistent with earlier work in which ATF6-knockout MEFs had lower CHOP levels until 12 h of exposure to thapsigargin, whereas at later time points CHOP levels were higher compared with WT (Diedrichs et al., 2018). Given the importance of ATF6 in the regulation of CHOP activation dynamics as well as cytoprotective proteins such as BiP (Vitale et al., 2019), ATF6 is also expected to play a role in the switch between adaptive to cellular adversity, especially in realistic scenarios with repeated exposure to chemicals. Indeed, it has been reported that ATF6 plays a role in the protection against chronic ER stress using ATF6 knockout mice and repeated exposures (Wu et al., 2007).

In conclusion, by combining high-throughput confocal imaging and ODE modeling, we captured the dynamics and role of individual components within the UPR, particularly pinpointing the importance of ATF6 in CHOP activation dynamics. Since the UPR plays an important role in both drug-induced toxicity as well as the development of drug resistance in cancer, improved insight in UPR signaling dynamics in relation to cell fate is important.

### Limitations of the Study

Using a combined experimental and computational modeling study of UPR signaling, we showed that ATF6 has an important role in shaping the dynamic pattern of CHOP activity, thus likely affecting cell fate decisions under ER stress. However, we neither mathematically described the relation between UPR activity and cell fate, nor did we investigate experimentally whether cell fate decisions are indeed affected by ATF6. Moreover, our findings are based on a single cell line and only on *in vitro* observations, hence the response may be different in *in vivo* scenarios. Finally, we do not know whether our observations hold for other UPR-invoking compounds and whether our model is able to describe UPR dynamics for such compounds, including their potential adversity.

## Methods

All methods can be found in the accompanying Transparent Methods supplemental file.

## Data and Code Availability

We put the python script to simulate the developed computational model as the Supplemental Information.

## Supplemental Information

Document S1. Transparent Methods, Figures S1–S11, and Table S1
