# Supplementary material for: ATF6 Is a Critical Determinant of CHOP Dynamics during the Unfolded Protein Response
Source: iScience. 2020 Jan 23;23(2):100860. doi: 10.1016/j.isci.2020.100860 (PMC7005498; doi:10.1016/j.isci.2020.100860)
Supplement: Document S1. Transparent Methods, Figures S1–S11, and Table S1 [file mmc1.pdf]

iScience, Volume *23*

Supplemental Information

ATF6 Is a Critical Determinant of CHOP Dynamics during the Unfolded Protein Response

Huan Yang, Marije Niemeijer, Bob van de Water, and Joost B. Beltman

Supplemental data items

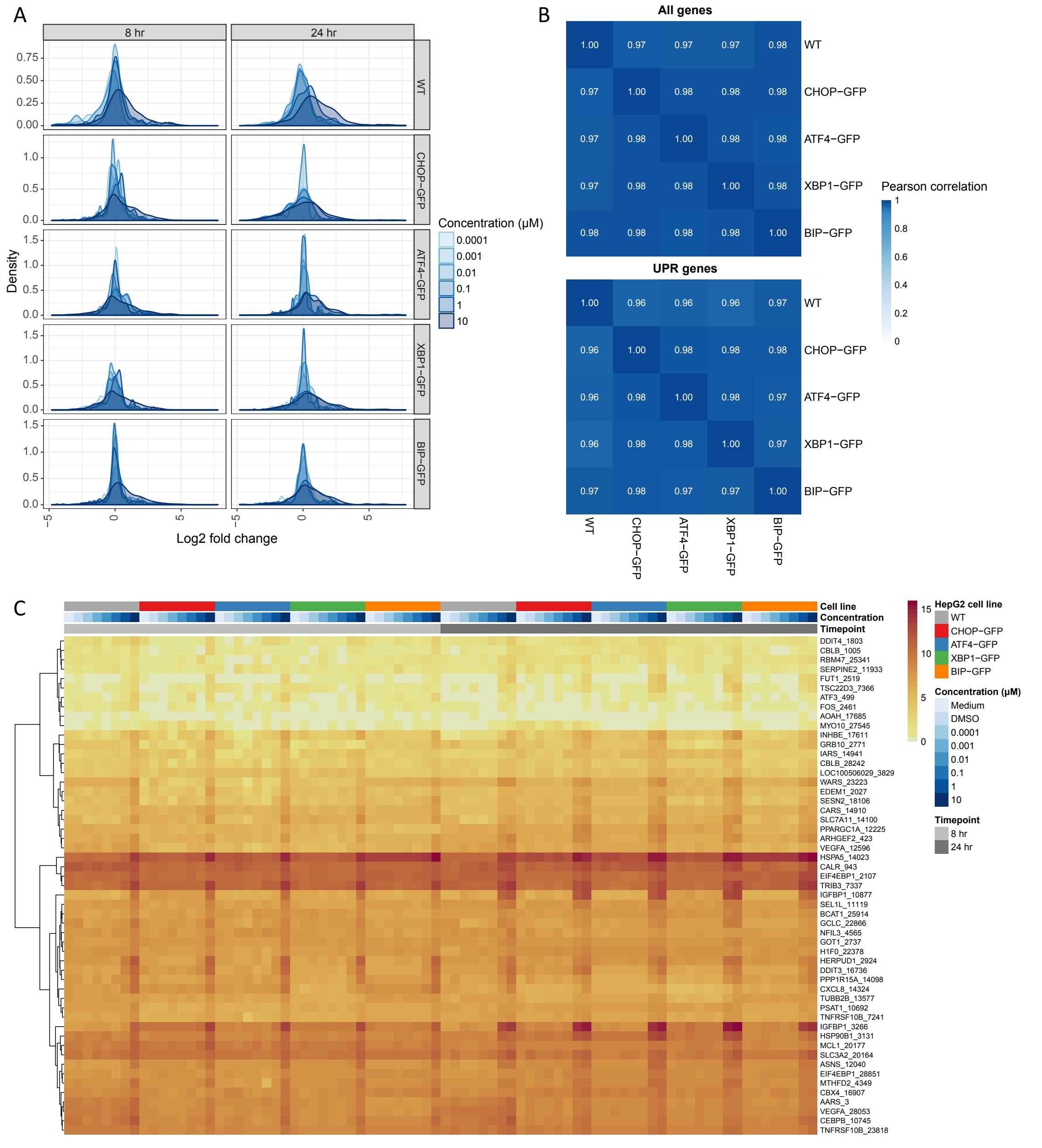

Figure S1: **Transcriptomic profiling of HepG2 UPR BAC-GFP reporters, related to Figure 2**. **A**: Distribution of log2 fold changes (compared to DMSO solvent control) of UPR-related genes across the indicated concentration range of tunicamycin for each HepG2 cell line (WT, CHOP-GFP, ATF4-GFP, pXBP1(S)-GFP and BiP-GFP) at 8 and 24h exposure time. **B**: Pearson correlation matrix of different HepG2 cell lines based on either all genes (top panel) or UPR-related genes (lower panel). Correlations are the mean of correlations between cell lines at each concentration of tunicamycin. **C**: Heatmap of log2 normalized counts of UPR-related genes (selection based on upper quantile of log2 fold changes of all UPR-related genes at 10 μM) for each HepG2 cell line exposed to the indicated concentration range of tunicamycin either for 8 or 24h. Hierarchical clustering of genes based on euclidean distance.

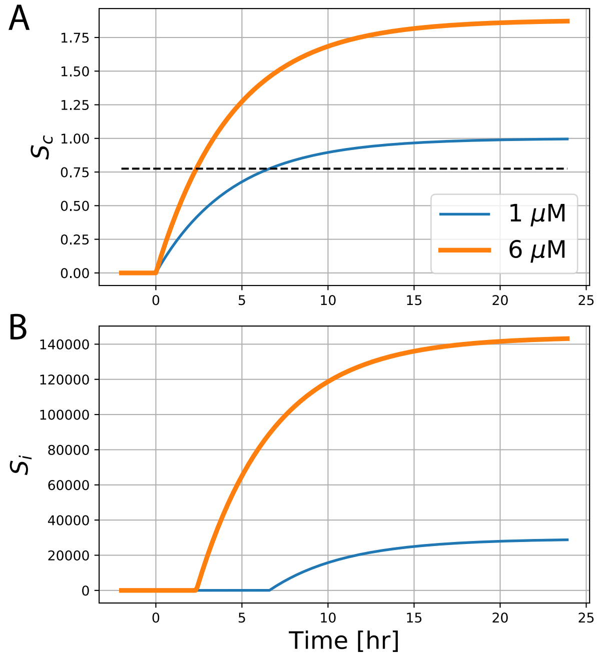

Figure S2: **Modeled pharmacokinetics of tunicamycin exposure, related to Figure 2 and 3**. **A**: effective intra-cellular concentration of tunicamycin S_c over time, **B**: exposure-related stressor S_i (unfolded proteins due to tunicamycin) which acts as input to the UPR signaling network.

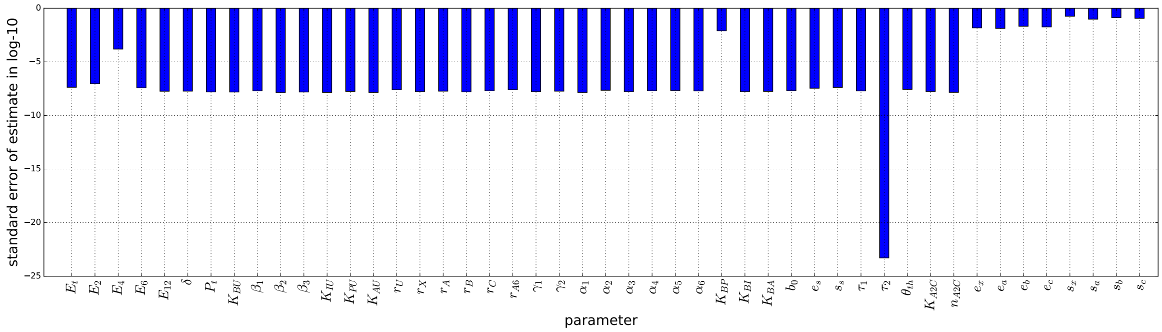

Figure S3: **Standard errors of model parameter estimates, related to Figure 3**. The standard errors were approximated via a Hessian-based approach and are presented in log10 scale.

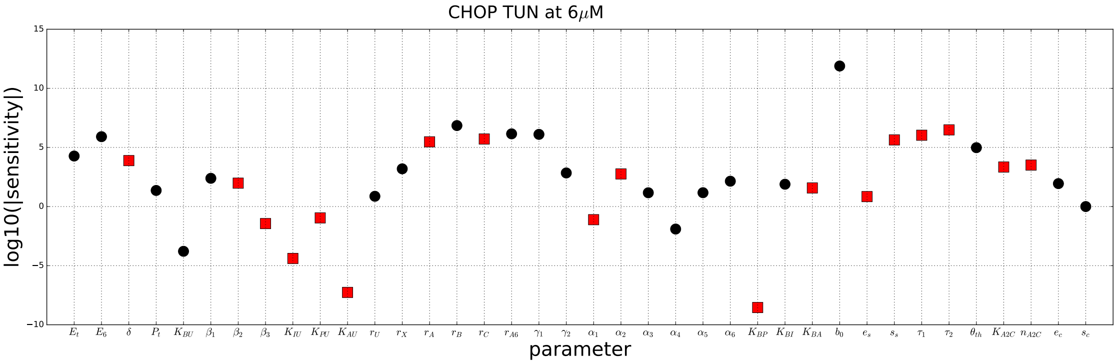

Figure S4: **Parameter sensitivity analysis of CHOP expression, related to Figure 3**. In the sensitivity analysis, we considered the sensitivity of CHOP expression at 16 hours after exposure to 6μM of tunicamycin. Parameters positively affecting CHOP are shown in black, while parameters negatively affecting CHOP are shown in red.

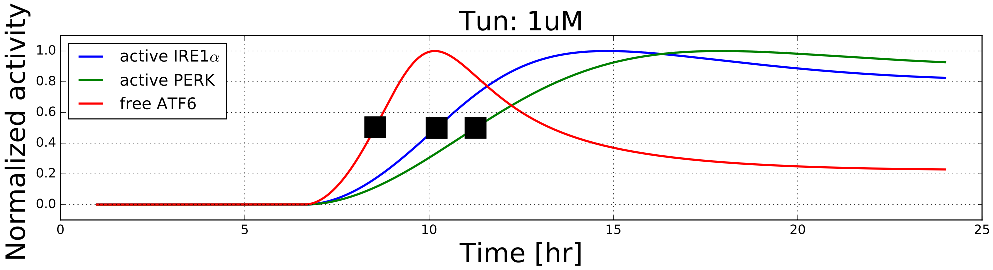

Figure S5: **Predicted dynamics of the three UPR sensors upon tunicamycin exposure, related to Figure 4**. Plot of the dynamics of the sensors IRE1α (blue), PERK (green), and ATF6 (red), after normalization to their maximally obtained value during the studies time period. Black squares indicate the moment at which sensor activity is half-maximal.

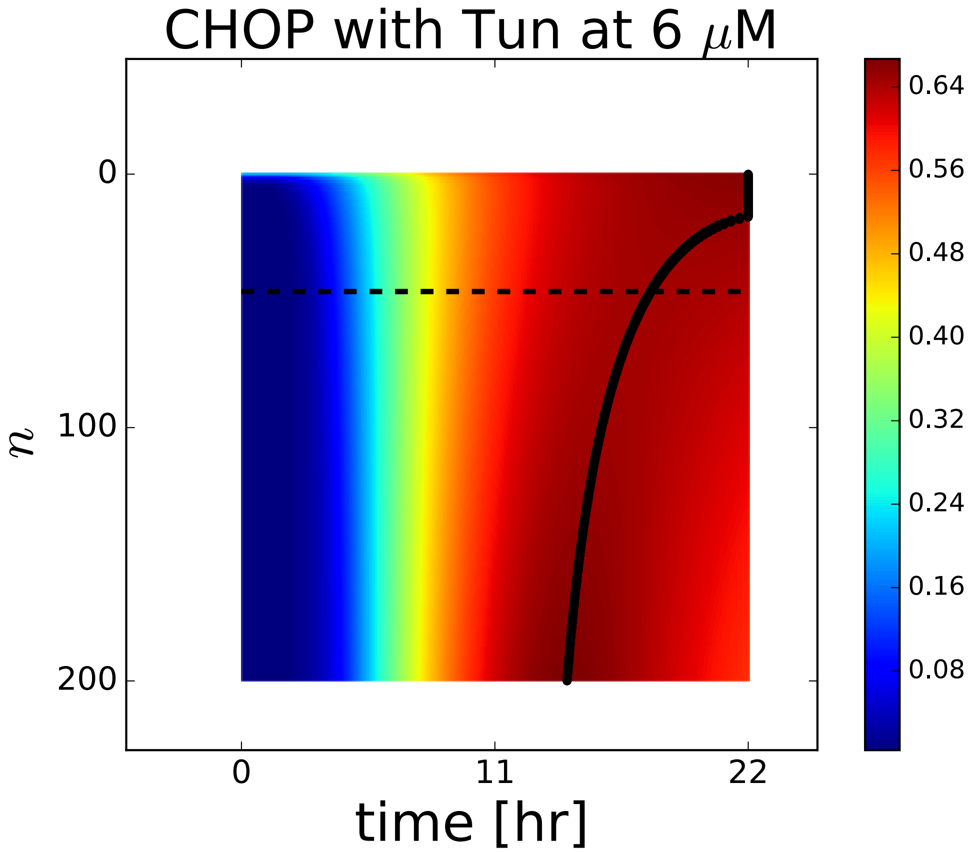

Figure S6: **Effect of n on CHOP upon exposure to 6 μM of tunicamycin, related to Figure 4**. Heat-map showing the temporal response of CHOP for a range of n values. The black solid line indicates the time point of maximal CHOP activity within the simulated time period. The black dashed line indicates the best fit value ($n = 46.32$).

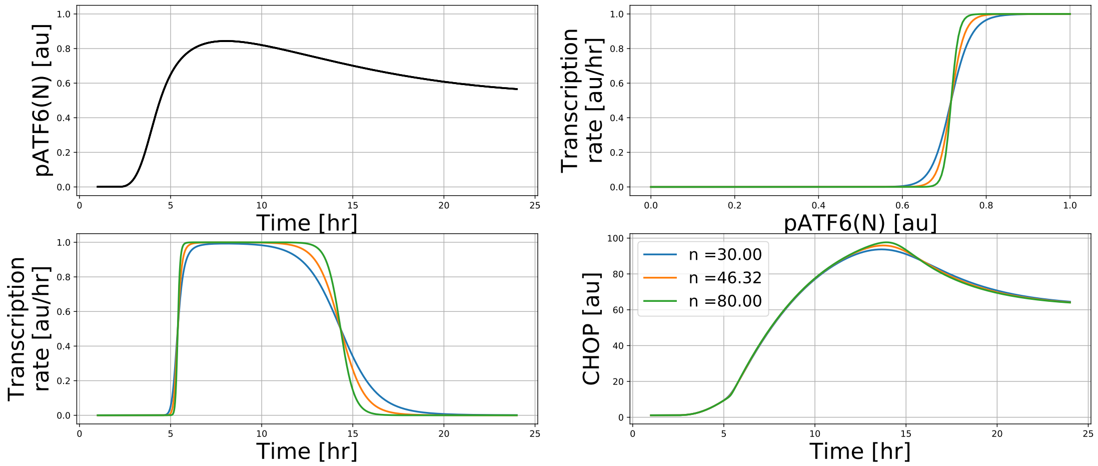

Figure S7: **Sensitivity of the CHOP response to the Hill coefficient describing the relation with pATF6(N), related to Figure 4**. For three values of n (including the estimated value of $n = 46.32$), we plot the pATF6(N) response over time (upper left panel), the relation between CHOP transcription and pATF6(N) level (upper right panel), the CHOP transcription rate due to pATF6(N) over time (lower left panel) and CHOP dynamics (lower right panel).

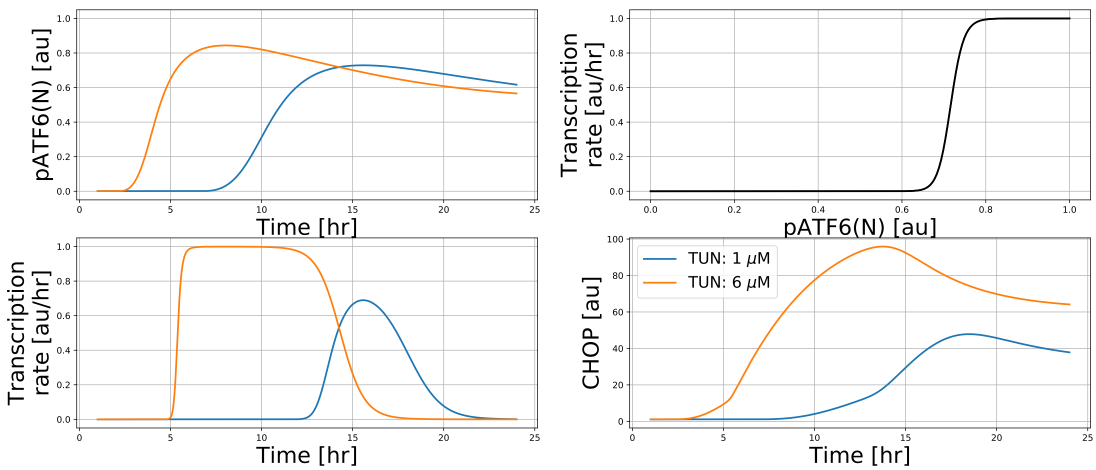

Figure S8: **Details of inner model states with respect to the CHOP response at different tunicamycin concentrations, related to Figure 4**. For two tunicamycin concentrations, we plot the pATF6(N) response over time (upper left panel), the relation between CHOP transcription and pATF6(N) level (upper right panel), the CHOP transcription rate due to pATF6(N) over time (lower left panel) and CHOP dynamics (lower right panel).

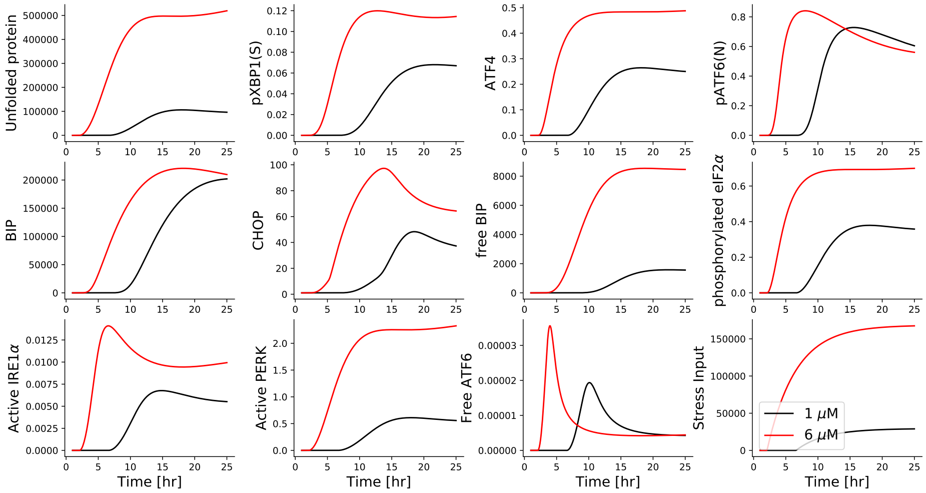

Figure S9: **Simulation of inner model states, related to Figure 3 and 4**. Dynamics of modeled UPR network components are shown upon exposure to tunicamycin at 1μM (black) and 6μM (red). Note that free ATF6 stands for activated ATF6 sensor, i.e., free uncleaved ATF6. Among the three branches, ATF4 tightly follows the dynamics of eIF2α_p and unfolded proteins.

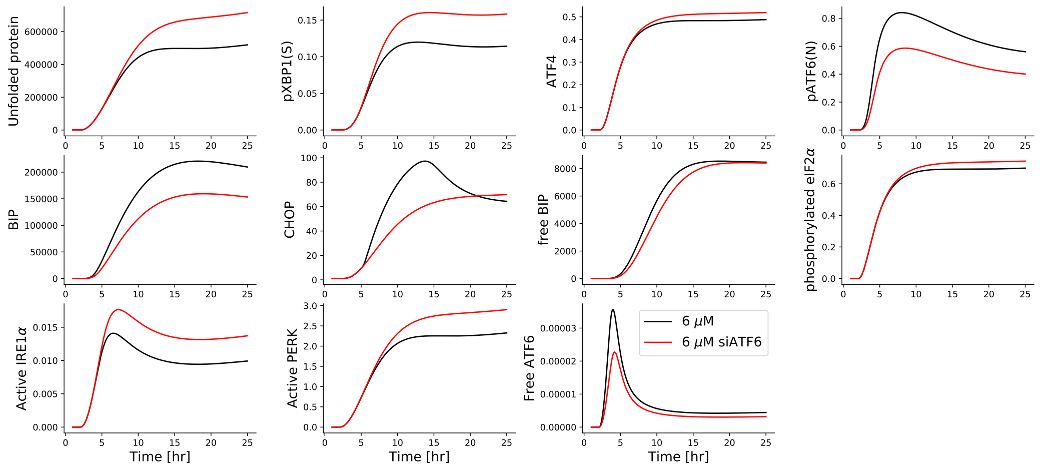

Figure S10: **Simulation of inner model states upon ATF6 knockdown, related to Figure 3 and 5**. Dynamics of modeled UPR network components upon exposure to 6μM tunicamycin, either with (red) or without (black) siATF6 treatment. Note that siATF6 results in lower BiP levels, which reduces the folding capacity. Hence, there are more unfolded proteins, which induces more ATF4 and pXBP1(S), in the long run leading to slightly higher CHOP levels compared to a setting without siATF6.

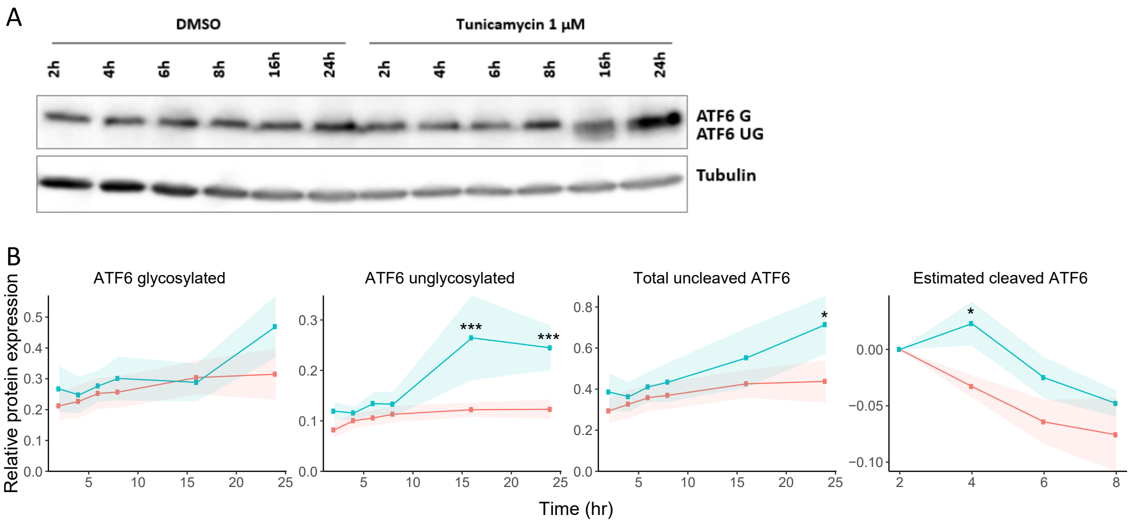

Figure S11: **Quantification of ATF6 forms after treatment of 1 μM tunicamycin, related to Figure 6**. **A**: Western blot of uncleaved ATF6 (G = glycosylated, UG = unglycosylated) measured in HepG2 WT cells at 2, 4, 6, 8, 16 or 24 hours after exposure to tunicamycin (1 μM). Tubulin protein expression was used as protein loading control. **B**: Quantified protein expression of ATF6 forms from three biological replicates after protein loading correction using tubulin (symbols and shaded area represent mean $\pm$ SD). Cleaved ATF6 was estimated based on the difference between total uncleaved ATF6 at 4, 6, or 8h and the total ATF6 at 2h.

Table S1: Model parameters, their units, their estimated values (± 95% confidence interval) and the boundary values used during the estimation procedure, related to Figure 3, 4, 5 and 6. For the rationale behind the choice of boundary values see the section on parameter ranges.

Parameter	unit	description	estimated $\boldsymbol{\theta_1}$	estimated $\boldsymbol{\theta_2}$± 95% CI	lower boundary	upper boundary
E_t	-	general translation rate (from mRNA to unfolded protein)	2.21e+00	2.00e+00 ± 8.45e-08	1.0	200.0
E_2	au	effective exposure at 2 μM	1.16e+00	1.22e+00 ± 1.80-e07	1.0	20.0
E_4	au	effective exposure at 4 μM	1.55e+00	1.67e+00± 3.10e-04	1.0	20.0
E_6	au	effective exposure at 6 μM	1.88e+00	2.07e+00 ± 7.45e-08	1.0	20.0
E_{12}	au	effective exposure at 12 μM	2.11e+00	2.48e+00± 3.62e-08	1.0	20.0
δ	au/hr	BiP-mediated folding rate	1.84e+01	1.96e+01 ± 3.70e-08	0.10	200.0
P_t	-	total amount of PERK	1.87e+01	8.36e+00 ± 3.12e-08	1.0	2.0e5
K_{BU}	au	Michaelis-Menten constant for dissociation of BiP and unfolded proteins	1.24e+07	1.24e+07 ± 3.07e-08	1e3	1e10
β_1	au/hr	IRE1α-dependent formation rate of XBP1	4.90e+00	2.75e+00 ± 3.90e-08	1.0e-8	2.0e8
β_2	au/hr	PERK-dependent ATF4 formation rate	1.52e+01	4.35e+00 ± 2.67e-08	1.0e-8	2.0e8
β_3	au/hr	ATF6-dependent ATF6f formation rate	-	1.18e+04 ± 3.08e-08	1.0e-8	2.0e8
K_{IU}	au	Michaelis-Menten constant for dissociation of IRE1α and unfolded proteins	9.57e+06	9.56e+06 ± 2.75e-08	1e1	1e10
K_{PU}	au	Michaelis-Menten constant for dissociation of PERK and unfolded proteins	1.35e+06	1.35e+06 ± 3.42e-08	1e1	1e10
K_{AU}	au	Michaelis-Menten constant for dissociation of ATF6 and unfolded proteins	-	1.08e+09 ± 2.71e-08	1e1	1e10
r_U	1/hr	degradation rate of unfolded proteins	4.07e-02	2.02e-08 ± 4.95e-08	1.0e-8	2.0e3
r_X	1/hr	degradation rate of XBP1	5.16e-01	2.34e-01± 3.29e-08	1.0e-8	2.0e3
r_{A_4}	1/hr	degradation rate of ATF4	4.95e+00	6.26e+00± 3.68e-08	1.0e-8	2.0e3
r_B	1/hr	degradation rate of BiP	2.58e-01	1.52e-01± 3.14e-08	1.0e-8	2.0e3
r_C	1/hr	degradation rate of CHOP	9.95e-01	2.83e-0± 3.92e-08	1.0e-8	2.0e3
r_{A_6}	1/hr	degradation rate of ATF6f	-	1.05e-0± 4.96e-08	1.0e-8	2.0e3
γ_1	au/hr	basal BiP transcription rate	1.13e+00	6.25e-01± 3.26e-08	0	2e2
γ_2	au/hr	basal CHOP transcription rate	3.29e-01	2.96e-01± 3.68e-08	0	2e2
α_1	1/hr	XBP1-mediated BiP transcription rate	8.14e+04	8.14e+0± 2.68e-08	0	1e6
α_2	1/hr	ATF4-mediated BiP transcription rate	2.97e+03	2.60e+02± 4.43e-08	0	1e6
α_3	1/hr	XBP1-mediated CHOP transcription rate	3.15e+02	3.17e+01± 3.26e-08	0	1e6
α_4	1/hr	ATF4-mediated CHOP transcription rate	2.86e+02	2.88e+01± 3.94e-08	0	1e6
α_5	1/hr	ATF6f-mediated BiP transcription rate	-	5.09e+04± 4.08e-08	0	1e6
α_6	au/hr	ATF6f-mediated CHOP transcription rate	-	1.39e+01± 3.87e-08	0	1e6
K_{BP}	au	Michaelis-Menten constant for dissociation of BiP and PERK	5.03e+07	5.03e+07± 1.56e-02	0	1e9
K_{BI}	au	Michaelis-Menten constant for dissociation of BiP and IRE1α	8.81e+02	1.92e+03± 3.27e-08	0	1e9
K_{BA}	au	Michaelis-Menten constant for dissociation of BiP and ATF6	-	7.81e+01± 3.45e-08	0	1e9
b_0	au/hr	basal production rate of ATF4	2.91e-06	2.41e-06± 4.00e-08	0	2e2
e_s	au/hr	factor scaling the effective intra-cellular concentration to unfolded proteins	1.31e+05	1.31e+05± 6.88e-08	1e-3	2e7
s_s	au/hr	net production rate of unfolded proteins independent of translation attenuation and exposure	-1.870	-2.00 ± 8.01e-08	-20.0	2e3
τ_1	1/hr	time constant describing initial increase in stressor	2.47e-01	2.26e-01± 3.87e-08	1e-15	5.0
τ_2	1/hr	time constant describing stressor decay	8.90e-03	8.45e-15± 1.01e-23	1e-15	5.0
θ_{th}	au	threshold for stressor levels that activate signaling	7.37e-01	7.75e-01 ± 5.29e-08	0.0	1.0
K_{A2C}	au	ATF6f level at which ATF6f-dependent CHOP transcription is half-maximal	-	7.17e-01± 3.32e-08	1e-8	1e4
n	-	cooperativity in ATF6f-dependent CHOP transcription Hill kinetics	-	4.63e+01± 2.86e-08	1e-2	1.0e2
e_{XBP1}	au	GFP scaling factor for XBP1 reporters	5.64	6.60± 2.94e-02	1.0e-7	1.0e2
e_{ATF4}	au	GFP scaling factor for ATF4 reporters	2.28e-01	1.23e+00± 2.58e-02	1.0e-7	1.0e2
e_{BIP}	au	GFP scaling factor for BiP reporters	1.13e-05	3.68e-06± 4.12e-02	1.0e-7	1.0e2
e_{CHOP}	au	GFP scaling factor for CHOP reporters	1.45e-02	7.75e-03± 3.60e-02	1.0e-7	1.0e2
s_{XBP1}	au	GFP offset for XBP1 reporters	5.20e-04	5.65e-04 ± 3.55e-01	-1.0e2	1.0e2
s_{ATF4}	au	GFP offset for ATF4 reporters	3.54e-02	4.19e-02± 1.90e-01	-1.0e2	1.0e2
s_{BIP}	au	GFP offset for BiP reporters	1.09e-01	7.81e-02± 2.61e-01	-1.0e2	1.0e2
s_{CHOP}	au	GFP offset for CHOP reporters	-7.10e-04	-3.89e-03 ± 2.26e-01	-1.0e2	1.0e2

Transparent Methods

Experimental details

Cell culture

HepG2 human hepatocellular carcinoma cells were purchased at American Type Culture Collection (ATCC, Wesel, Germany). To capture the induction of key proteins of the UPR, CHOP, ATF4, BiP and pXBP1(S) were GFP-tagged using a bacterial artificial chromosome (BAC) recombineering approach (Poser et al., 2008; Wink et al., 2014; Hendriks et al., 2011; Wink et al., 2017; Hiemstra et al., 2016). Hereby, stable HepG2 GFP-BAC reporter cell lines were established expressing protein-GFP fusions under control of the endogenous promoter for each gene. HepG2 cells were cultured in Dulbecco's Modified Eagle Medium (DMEM) containing 10% (v/v) fetal bovine serum (FBS), 25 U/mL penicillin and 25 μg/mL streptomycin at 37°C and 5% CO_2, and were used until passage 20. Cells were plated using a density of 70.000 to 140.000 cells/cm^2 when grown for 3 to 5 days.

Chemicals and antibodies

Tunicamycin was purchased at Sigma (Zwijndrecht, The Netherlands) which was dissolved in dimethylsulfoxide (DMSO) from BioSolve (Valkenswaard, The Netherlands) and stored at -20°C until usage. The maximum solvent end concentration of DMSO was at most 0.2% (v/v) to minimize the effect of the solvent itself. For western blotting, antibodies were used against CHOP, ATF4, pXBP1(S) and ATF6 from Cell Signaling (Bioké, Leiden, The Netherlands), BiP from BD Biosciences (Vianen, The Netherlands) at a dilution of 1:1000, and Tubulin from Sigma (Zwijndrecht, The Netherlands) at a dilution of 1:5000.

RNA interference

siRNA-mediated transient silencing of genes of interest in HepG2 cells was done using a reverse transfection approach. Prior to transfection, siGENOME SMARTpool siRNAs from Dharmacon (Eindhoven, the Netherlands) were mixed with INTERFERin from PolyPlus (Leusden, the Netherlands) for 10 minutes to allow for complex formation. Hereafter, siRNA mix, resulting in a 50 nM siRNA and 0.3% INTERFERin end concentration, together with cells at a density of 78.000 cells/cm^2 were added to each well. As control, mock (only INTERFERin) and siRNA scrambled non-targeting control was employed. At 24 hours post-transfection, medium was refreshed. siRNA-silenced cells were evaluated at 72 hours post transfection or exposed to compounds to assess the effect of the knockdown on drug-induced ER stress response activation.

Confocal Microscopy

Cells were plated in SCREENSTAR 96 wells or μClear 384 wells plates from Greiner Bio-One (Alphen aan den Rijn, The Netherlands) at the earlier mentioned cell densities. Prior to confocal microscopy imaging, cells were stained with 100 ng/mL Hoechst33342 for a minimum of 30 minutes to allow for nuclei visualization and cell tracking. To measure the induction of BAC-GFP intensity, cells were imaged live using an automated Nikon TiE2000 confocal microscope (Nikon, Amsterdam, The Netherlands) including an automated xy-stage, Perfect Focus System and lasers at wavelength 408, 488, 561 and 647nm. Cells were kept at 37°C and 5% CO2 humidified atmosphere during imaging.

Image Analysis

Segmentation and quantification of the GFP intensity was done using CellProfiler version 2.1.1 (Broad Institute Cambridge, USA) using analysis modules described previously (Wink et al., 2014; Niemeijer et al., 2018). In brief, nuclear segmentation based on Hoechst signal was done using an in-house constructed watershed masking algorithm (Di et al., 2012). The propagation

segmentation method based on GFP signal was used for cytoplasm segmentation. GFP intensity was measured in the nucleus as well as in the cytoplasm. For subsequent analysis, Rstudio version 1.0.153 (Boston, USA) was used. For alignment of the data acquired around discrete time points (1,2,..., 24 hours), we employed cubic interpolation of the GFP intensity such that standard deviations can be estimated from the individual replicates, which are integrated into the cost function for parameter estimation (see Supplementary text about single-cell data analysis for details).

TempO-seq transcriptomics

To assess mRNA levels, cells were seeded in 96 wells plates from Corning (Amsterdam, The Netherlands) using a density of 156.000 cells/cm^2. After compound exposure the following day, cells were washed with 1x PBS and lysed using 50 μL per well in 1x BNN lysis buffer from BioSpyder (Carlsbad, USA). After a 15 minute incubation period at room temperature, lysates were frozen at -80°C. As internal control, 0.05 μg/μL Universal Human RNA Reference (MAQC) in 1x BNN lysis buffer was used. Lysates were sent to and analyzed by BioSpyder Technologies Inc. (Carlsbad, USA) using the TempO-seq technology (Yeakley et al., 2017) of a targeted gene set consisting of the S1500+ gene list (Mav et al., 2018). In brief, a pair of detector oligos hybridized to its specific target mRNA leading to oligo pair ligation. This was followed by PCR amplification of ligated pairs of oligos incorporating also a sample-barcode and adaptors, which was subsequently sequenced. Alignment of raw reads was done using the TempO-seqR package (BioSpyder Technologies Inc., Carlsbad, USA). Read counts were normalized using the DESeq2 R package (Love et al., 2014) and log2 transformed. UPR-related genes were defined by selecting target genes of transcription factors ATF4, ATF6, pXBP1(S) and DDIT3 that were based on DoRothEA (Discriminant Regulon Expression Analysis) v2 (Garcia-Alonso et al., 2018) using confidence level A to D and that were present in the S1500+ geneset.

Western blot analysis

For western blot analysis, samples were collected after two wash steps with ice-cold 1x PBS by adding 1x sample buffer supplemented with 10% v/v β-mercaptoethanol and stored at -20°C. Prior to loading, samples were heat-denatured at 95°C for 10 minutes. Proteins were separated on SDS-page gels using 120 volt and transferred to polyvinylidene difluoride (PVDF) membranes at 100 volt for 2 hours. After blocking using 5% ELK, membranes were stained with primary and secondary HRP- or Cy5-conjugated antibodies diluted in 1% bovine serum albumin (BSA) in tris-buffered saline (TBS)-0.05%Tween20. Thereafter, Enhanced Chemiluminescent (ECL) western blotting substrate from Thermo Scientific (Bleiswijk, The Netherlands) enabled to visualize the HRP-conjugated antibody staining using the Amersham Imager 600 from GE Healthcare (Eindhoven, The Netherlands). Protein expression was quantified using ImageJ version 1.51h (National Institutes of Health, USA) and normalized to tubulin protein expression.

Statistics

Confocal microscopy data from three biological replicates is represented as the mean ± SE. TempO-seq gene expression data was represented either as log2 normalized counts ± SE or as log2 fold changes with standard error calculated using the DESeq2 R package (Love et al., 2014). Significance was determined with the Wald test and Benjamini Hochberg correction using the DESeq2 R package (Love et al., 2014). Significance for TempO-seq gene expression data was determined at three threshold levels ($*p_{adj} < 0.05$, $**p_{adj} < 0.01$, $***p_{adj} < 0.001$). Western blot data for ATF6 quantification originated from three biological replicates and were represented as the mean ± SE. Here, significance levels were calculated using unpaired Student's t test with Benjamini Hochberg multiple testing correction, represented as $*p_{adj} < 0.1$, $**p_{adj} < 0.05$, $***p_{adj} < 0.01$. Processing and visualization of all data was done using Rstudio version 1.0.153 (Boston, USA) in combination with R 3.4.1 and the following R packages: ggplot2 (Wickham, 2010), RColorBrewer

(Neuwirth, 2014), data.table (Dowle et al., 2018), dplyr (Wickham et al., 2011), tidyr (Wickham, 2017), reshape2 (Zhang, 2016), scales, stats and splines.

Computational modeling

UPR model construction and simulation

We built a dynamic model of the UPR signaling network with six state variables: unfolded protein (U), pXBP1(S) (X), ATF4 (A_4), ATF6 fragment(A_6), BiP (B), and CHOP (C). These states represent concentrations of molecules per cell and their dynamics are mathematically described by a set of ordinary differential equations. The equations obey kinetics of biochemical reactions including mass-action, Michaelis-Menten or Hill kinetics. We simplified the model in a similar way as (Trusina et al., 2008; Diedrichs et al., 2018) with quasi-steady state assumptions for association or dissociation of complexes and modulation effects. Furthermore, we took multiple conservation terms into account in order to reduce the number of state variables. We extended the available model of (Trusina et al., 2008) by incorporating ATF4 and CHOP. Furthermore, because ATF6 is proteolytically processed but this is not the case in the XBP1 branch (Ye et al., 2000), we considered the possibility that ATF6 and XBP1 need to be assigned different parameters (e.g., their degradation rates) to allow these branches to respond differently. To take the pharmacokinetics of the exposure into account, we modeled the intra-cellular concentration of tunicamycin as a function with two exponents, which represents the analytical solution to a linear system for two compartments (i.e., the medium in which cells reside and intra-cellular spaces).

The set of ODEs is mathematically represented as

$$\dot{\mathbf{x}}(t) = \mathbf{f}(\mathbf{x(t)}, \mathbf{u(t)}, \theta), \tag{1}$$

where $x(t)$ stands for the six state variables of the dynamic system, $u(t)$ is the input function, and θ contains the system parameters. The dynamics of the UPR state variables are described by:

$$\begin{cases} \dot{U} = f_1(x), \\ \dot{X} = f_2(x), \\ \dot{A}_4 = f_3(x), \\ \dot{A}_6 = f_4(x), \\ \dot{B} = f_5(x), \\ \dot{C} = f_6(x), \end{cases} \tag{2}$$

with initial condition

$$\mathbf{x}_0 = (U_0, X_0, A_{4,0}, A_{6,0}, B_0, C_0)^{\cdot} \tag{3}$$

In the following the right hand sides of equations (2) are provided for each state. Our modeling work follows (Trusina et al., 2008) assuming a quasi steady-state for sensors which can bind to BiP or to unfolded proteins. In addition, we incorporated the ATF6 branch and the downstream molecules ATF4 and CHOP (Trusina et al., 2008). This allows to integrate all experimental data obtained from our GFP reporter cell lines, i.e., pXBP1(S), ATF4, BiP and CHOP.

We subsequently describe all equations for the system states, starting with the unfolded protein U:

$$f_1(\mathbf{x}) = \frac{E_t}{1 + P_{act}} + s_s + S_i - \delta\, B_f - r_U\, U\,, \tag{4}$$

where E_t denotes the base rate of translation, i.e., the formation of peptides or unfolded proteins from mRNA which can be modulated by translation attenuation. S_i represents the rate of production of unfolded proteins due to the exposure-related stressor, which is described explicitly as

a function of time (see below). The parameter s_s represents a net folding/unfolding rate that is independent of BiP and of translation. Instead, it includes both the folding activity of chaperones other than BiP and unfolding activity of existing proteins. Because it represents a net effect, s_s can have a positive or negative value, depending on which process prevails. Unfolded proteins are removed by degradation, which occurs at rate r_U, or by their folding following binding to the chaperone BiP, which occurs at rate δ. The latter process depends on the amount of free form of BiP, which is given by

$$B_f = \frac{U}{U + K_{BU}} B , \tag{5}$$

where K_{BU} is the amount of unfolded proteins for which half of the BiP molecules is present in free form. Inhibition of translation is modeled by modification of the E_t term, where P_{act} denotes the active form of PERK and is given by

$$P_{act} = P_t \, (U/K_{PU})/(1 + B_f/K_{BP} + U/K_{PU}) , \tag{6}$$

where P_t is the effective/net amount of PERK, and K_{PU} and K_{BP} are Michaelis-Menten parameters describing the affinity of the complexes PERK:UP and BiP:UP, respectively.

The amount of spliced XBP1 is described by:

$$f_2(\mathbf{x}) = \beta_1 \, I_{act} - r_X \, X . \tag{7}$$

Here, β_1 represents the XBP1 splicing rate, which depends on the amount of active IRE1α. The latter is represented by I_{act} and is given by:

$$I_{act} = (U/K_{IU})/(1 + B_f/K_{BI} + U/K_{IU}) , \tag{8}$$

where K_{IU} and K_{BI} are Michaelis-Menten parameters describing the affinity of the complexes IRE1α:UP and BiP:IRE1α, respectively. Spliced XBP1 is degraded at rate r_X.

The amount of ATF4 is described by:

$$f_3(\mathbf{x}) = b_0 + \beta_2 \, eIF2\alpha_p - r_{A_4} \, A_4, \tag{9}$$

where r_{A_4} denotes the degradation rate of ATF4, b_0 indicates its basal production rate, and β_2 is the additional production rate of ATF4 due to $eIF2\alpha_p$, where eIF2α_p is the fraction of phosphorylated eIF2α that obeys:

$$eIF2\alpha_p = 1 - eIF2\alpha_{up} = 1 - \left(1 + \frac{P_t \, U}{K_{PU} + B_f \, K_{PU}/K_{BP} + U}\right)^{-1} , \tag{10}$$

where $eIF2\alpha_{up}$ denotes the fraction of unphosphorylated eIF2α. Note that the total amount of $eIF2\alpha$ (phosphorylated and unphosphorylated) is considered to be conserved.

The amount of pATF6(N) is described by:

$$f_4(\mathbf{x}) = \beta_3 \, A_{6,act} - r_{A_6} \, A_6, \tag{11}$$

where $A_{6,act}$ is the activated sensor (i.e., the free form of ATF6, which is not the same as pATF6(N)), which obeys

$$A_{6,act} = (U/K_{AU})/(1 + B_f/K_{BA} + U/K_{AU}) . \tag{12}$$

As before, K_{AU} and K_{BA} are Michaelis–Menten parameters representing the affinity of the complexes ATF6:UP and BiP:ATF6, respectively.

The amount of BiP is described by:

$$f_5(\mathbf{x}) = \gamma_1 + \alpha_1 \, X + \alpha_2 \, A_4 + \alpha_5 \, A_6 - r_B \, B , \tag{13}$$

where γ_1 is the basal production rate of BiP, r_B is the degradation rate of BiP, and α_1, α_2, α_5 represent the additional BiP production rate due to activity of pXBP1(S), ATF4 and pATF6(N), respectively.

The amount of CHOP is described by:

$$f_6(\mathbf{x}) = \gamma_2 + \alpha_3\, X + \alpha_4\, A_4 + \alpha_6\, A_{6,hill} - r_C\, C, \tag{14}$$

where γ_2 is the basal production rate of CHOP, r_C is the degradation rate of CHOP, and α_3, α_4, α_6 represent the additional CHOP production rate due to activity of pXBP1(S), ATF4 and pATF6(N), respectively. $A_{6,hill}$ describes the contribution of pATF6(N) to the CHOP transcription rate with a Hill function:

$$A_{6,hill} = \frac{A_6^n}{A_6^n + K_{A2C}^n}\,, \tag{15}$$

where n and K_{A2C} are the exponent and threshold in the Hill-function. Note that because there is not a clear peak in the dynamics of pXBP1(S) or ATF4 shown in Fig. 2D in the main text, a Hill function with an exponent larger than one is not needed to describe the effect of pXBP1(S) and ATF4 on CHOP (i.e., the fitting performance is not improved). Hence, for the sake of simplicity, we only used a Hill function for pATF6(N).

In addition to the six state variables, the exposure-related stressor S_i is a dynamic variable whose kinetics do not depend on the other system states. The intra-cellular concentration of the applied compound (S_c) is described explicitly with the following pharmacokinetics (see the supplementary subsection "Describing cellular exposure with a two-compartment model" for details about its derivation):

$$S_c = E_i \left(e^{(-\tau_2 t)} - e^{(-\tau_1 t)} \right) H(t). \tag{16}$$

Here E_i represents the effective intra-cellular concentration of the applied compound, with the subscript i denoting the applied concentration in μM. For 1 μM, we set $E_1 = 1$; for the other four concentrations we assign four free parameters that are estimated (see Table S1). $H()$ stands for the Heaviside function. We consider the stressor to affect the signaling network only when a threshold θ_{th} is crossed. We describe this by the above discussed S_i, i.e., the effective rate at which unfolded proteins are formed due to the stressor:

$$S_i = e_s\,(S_c - \theta_{th}) H(S_c - \theta_{th}), \tag{17}$$

where e_s scales the effective intra-cellular concentration of tunicamycin to unfolded proteins. At equilibrium, the total production rate of unfolded proteins is $\frac{E_t}{1+P_{act}} + s_s$.

For each GFP-reporter cell line, we introduce scaling and offset parameters denoted as e_{GFP} and s_{GFP}, respectively. Those two parameters transform the state in the ODE to the observable. For example, for ATF4 we formulated the observable A_4^o as

$$A_4^o = e_{atf4}\, A_4 + s_{atf4}. \tag{18}$$

Hence, to map the concentrations of the proteins to the GFP intensities, we introduce eight parameters for the four UPR cell lines: e_{xbp1}, s_{xbp1}, e_{atf4}, e_{chop}, s_{xbp1}, s_{atf4}, s_{bip}, and s_{chop}.

All model simulations were conducted in python 2.7.14.

Model calibration and model selection

We fitted our models to the quantified dynamics of reporter cell lines, using the maximum likelihood approach to estimate parameters. Given the nonlinear nature of the model, multiple local optima of parameters could exist in the likelihood landscape. To find the global optimum, we employed a Monte Carlo method with multiple starting values. We generated a set of $N_s = 1000$ starting values $\{\boldsymbol{\theta_s}\}$ in $\boldsymbol{\Theta}$ using Latin hypercube sampling (McKay et al., 2000). We listed the employed boundaries of the parameters in Table S1. For each starting value, we use the Trust-Region-Reflective-Newton method to obtain the local minimum $\boldsymbol{\theta_f}$ (Coleman & Li, 1996). For a

robust and efficient estimation, we incorporate the sensitivity equation (Raue et al., 2013) and a steady state constraint (Rosenblatt et al., 2016) into our local optimization. After applying this local optimization for all starting values, we take the estimate $\hat{\boldsymbol{\theta}} = \boldsymbol{\theta_f}$ with the minimal negative log-likelihood. Numerical optimization relied on the python package scipy. To quantify the uncertainty of parameter estimates, we applied a Hessian-based approach to explore the likelihood around the estimates. We quantified this as a 95% confidence interval of the estimates (Raue et al., 2009) (see details in the Supplement "Covariance matrix of estimates" and confidence intervals of the estimates in Table S1). Moreover, we performed a sensitivity analysis of the impact of single parameters on the CHOP level based on the maximum likelihood estimate, i.e., the most plausible set of parameters based on the measurements (see details in the Supplement "sensitivity analysis").

Plausible models are expected to give a good fit to observations with a relatively small value of the negative-log likelihood at $\hat{\boldsymbol{\theta}}$. We performed a likelihood-ratio-based test to evaluate the goodness of fit to the measurements, as for example applied in (García-Pérez & Alcalá-Quintana, 2015a,b) aiming to get insight into processes underlying temporal-order and simultaneity judgments by observers. Specifically, García-Pérez & Alcalá-Quintana (2015a) focused on a likelihood-ratio based approach to check goodness of fit and García-Pérez & Alcalá-Quintana (2015b) further incorporated the likelihood ratio into a Bayesian test when computing the ratio of two posterior distributions in order to derive a closed-form psychometric function about simultaneity judgments. In general, the test can be used to compare two models by the ratio of their likelihoods, denoted by $\triangle G$, and a p value is computed from a χ^2 distribution. In our case, we used this approach to evaluate whether the data are more compatible with separate incorporation of the ATF6 branch (with estimates $\boldsymbol{\theta_2}$) rather than with lumping ATF6 and XBP1 into a single branch (with estimates $\boldsymbol{\theta_1}$).

Modeling of knockdown conditions

We simulated the calibrated model by incorporating single knockdown perturbations with siRNA treatments. We focused on the knockdowns with siDDIT3 and siATF4, and siATF6, setting the knockdown efficiencies at the values estimated by the analysis of the TempO-seq data. To account for variability of knockdown efficiencies over experiments and over time from different assays, we varied the knockdown efficiency by 20% more or less than the reference value and simulated the model accordingly.

Here we describe how we model knockdown experiments by siRNA treatments. The dynamics of the mRNA can be described by the following differential equation:

$$\tau_m \frac{d}{dt}[\text{mRNA}] = \lambda_m + \text{tf(t)} - d_m \, [\text{mRNA}] \,, \tag{19}$$

where [mRNA] represents the amount of mRNA of interest, τ_m is the time constant of the mRNA, tf(t) is mRNA production rate due to TF activity, λ_m denotes the basal production rate, and d_m is the degradation rate of the mRNA. We consider knockdown of a gene of interest to increase the mRNA degradation rate compared to the control case. To study how this affects the protein dynamics over time, we first write the equation for the protein:

$$\tau_p \frac{d}{dt}[\text{protein}] = \lambda_p[\text{mRNA}] - d_p \, [\text{protein}] \,, \tag{20}$$

where [protein] represents the amount of protein of interest, τ_p is the time constant of the protein and d_p is the degradation rate of the protein.

Considering transcription to be much faster than (post-)translational processes, i.e. $\tau_m \ll \tau_p$, the mRNA will be at equilibrium, i.e., $[\text{mRNA}](t) = \frac{\lambda_m + \text{tf}(t)}{d_m}$.

Substitution of this relation into the translation step in (20) gives

$$\tau_p \frac{d}{dt}[\text{protein}] = \lambda_p \frac{\lambda_m}{d_m} + \lambda_p \frac{\text{tf(t)}}{d_m} - d_p \, [\text{protein}]. \tag{21}$$

Lumping $\frac{\lambda_m}{d_m}$ into λ_p^* and $\lambda_p \frac{\lambda_m}{d_m}$ into μ_p^* results in:

$$\tau_p \frac{d}{dt}[\text{protein}] = \mu_p^* + \lambda_p^* \text{tf} - d_p\,[\text{protein}]. \tag{22}$$

In this equation, μ_p^* and λ_p^* incorporate the effects of the increased mRNA degradation upon knockdown. Thus, these parameters are expected to decrease when cells are pre-treated with siRNA knockdowns. We define the knockdown efficiency e_{KD} as $1 - \frac{[mRNA]_{KD}}{[mRNA]}$, which equals $1 - \frac{d_m}{d_{m,KD}}$, where $d_{m,KD}$ is the mRNA degradation rate upon knockdown. Then we obtain $d_{m,KD} = \frac{d_m}{1-e_{KD}}$, which propagates into the parameters for protein formation as $\mu_{p,KD}^* = (1 - e_{KD})\,\mu_p^*$ and $\lambda_{p,KD}^* = (1 - e_{KD})\,\lambda_p^*$. In conclusion, the knockdowns can be simulated by decreasing the protein production rates with a multiplier based on the measured knockdown efficiency.

For ATF4, ATF6 and CHOP, for which we obtained knockdown efficiencies, we thus perturbed the production rates as follows: For siATF4, we set $\beta_2 = (1 - e_{KD})\hat{\beta_2}$ and $b_0 = (1 - e_{KD})\hat{b_0}$, where $\hat{\beta_2}$ and $\hat{b_0}$ are the estimated values in the absence of knockdown. For siATF6, we set $\beta_3 = (1 - e_{KD})\hat{\beta_3}$, where $\hat{\beta_3}$ is the estimated value in the absence of knockdown. For siDDIT3, we set $\gamma_2 = (1 - e_{KD})\hat{\gamma_2}$, $\alpha_3 = (1 - e_{KD})\hat{\alpha_3}$, $\alpha_4 = (1 - e_{KD})\hat{\alpha_4}$, and $\alpha_6 = (1 - e_{KD})\hat{\alpha_6}$, where $\hat{\gamma_2}$, $\hat{\alpha_3}$, $\hat{\alpha_4}$, and $\hat{\alpha_6}$ are the estimated values in the absence of knockdown.

Supplementary Details

Analysis of single-cell data

For the analysis of single-cell imaging data, we applied the following steps: First, for each of three biological replicates (each consisting of two pooled technical replicates), we calculated the geometric mean (denoted by I) based on GFP measurements for thousands of cells per image well at all time points and for all treatment conditions. Second, from this analysis we obtained the minimum (denoted by I_{min}) and maximum (denoted by I_{max}) of these geometric means with respect to all conditions and time points (separately for every cell line in every plate). We then applied min-max normalization to obtain the normalized intensity (denoted by I_N) each time point according to:

$$I_N = \frac{I - I_{min}}{I_{max} - I_{min}} \,. \tag{23}$$

Third, we interpolated the normalized means to the time points from 1h to 22h as described in the main text (note that some treatment conditions only had data before 23h). Finally, we took the arithmetic mean and standard deviation of the interpolated data for the biological replicates, which we used for further model fitting purposes.

Describing cellular exposure with a two-compartment model

To describe the exposure of cells to the chemical tunicamycin, we introduce a two-compartment model describing the concentrations of the chemical in the medium ($[C_1]$) and in the cells ($[C_2]$):

$$\begin{cases} \frac{d[C_1]}{dt} &= [D]\delta(t) - \tau_1[C_1] \,, \\ \frac{d[C_2]}{dt} &= \tau_1[C_1] - \tau_2[C_2] \,. \end{cases} \tag{24}$$

Here, τ_1 is the cellular absorption rate from the medium, τ_2 is the degradation rate of the chemical within cells and $[D]$ and $\delta(t)$ are the applied exposure and unit pulse input functions, respectively. To obtain the solution of the above set of ODEs, one can take a convolution: In general, for $g(t) = \int f(\tau)\, h(t-\tau)d\tau$, $g(t)$ is the output function, $f(t)$ is the input function and $h(t)$ is the transfer function of the linear system, which can be derived by Laplace transformation. In our case, the transfer functions for $[C_1]$ and $[C_2]$ are $\exp(-\tau_1 t)H(t)$ and $\exp(-\tau_2 t)H(t)$, respectively. For $[C_1](t)$ we then obtain the solution $[C_1](t) = [D]\exp(-\tau_1 t)H(t)$. Furthermore, for $[C_2](t)$ we obtain:

$$[C_2](t) = [D]\exp(-\tau_2 t)\left(\exp(-(\tau_2 - \tau_1)t)(\tau_2 - \tau_1)^{-1} - (\tau_2 - \tau_1)^{-1}\right) . \tag{25}$$

This simplifies to:

$$[C_2](t) = (\tau_2 - \tau_1)^{-1}\tau_1[D](\exp(-\tau_1 t) - \exp(-\tau_2 t)). \tag{26}$$

To avoid structural non-identifiability issues, we absorb the term $\tau_1(\tau_2 - \tau_1)^{-1}$ into the parameter e_s that scales the stressor (see Eq. (17) in main text), leaving us with:

$$[C_2](t) = [D](\exp(-\tau_1 t) - \exp(-\tau_2 t))H(t). \tag{27}$$

Activation of the three UPR sensors

Our calibrated model can be used to provide insight into the activation speed of the three UPR branches. We therefore quantified the moment at which the active forms of the sensors (active IRE1α, active PERK, and free ATF6) reach their half-maximal value, taking 1 μM of tunicamycin as a representative case (Fig. S5).

Effect of Hill coefficient on CHOP transcription

Because the estimated value of the Hill coefficient in the relation between $A_{6,hill}$ and A_6 (Eq. (15) in main text) is very high ($n = 46.32$), this implies a switch-like response of CHOP transcription with increasing pATF6(N), questioning the suitability of a lower n value. In the model, the sigmoid dependency of CHOP expression on pATF6(N) levels is complemented by linear dependencies on XBP1 (with parameter α_3) and on ATF4 (with parameter α_4) (Eq. (14) in main text). In order to understand the effect of the Hill exponent n on CHOP regulation, we varied n over a wide range (from 0 to 200) while keeping the other model parameters the same and plotted the predicted CHOP response in a two-dimensional heat-map with time on the horizontal axis and n on the vertical axis (Fig. S6). This analysis shows that an increase of the exponent beyond the calibrated value ($n = 46.32$) still has a clearly noticeable effect on the dynamics of CHOP. Consistent with sensitivity of the CHOP response to n, high values of n lead to a more pronounced peak in CHOP levels and an even more step-like response of CHOP transcription with pATF6(N) concentration and with time compared to low values of n (Figs. S7 and S8). As a side note, because CHOP in our model does not provide feedback to any of the other state variables, the Hill coefficient n will not affect the pATF6(N) concentration itself. Thus, this analysis shows that the value of the Hill exponent is important in determining the CHOP dynamics, especially around the time of its peak.

Contribution of pXBP1(S) and ATF4 to CHOP production

According to the model calibration, the coefficients describing pXBP1(S)-mediated and ATF4-mediated CHOP transcription respectively are $\alpha_3 = 31.77$ (pXBP1(S)), and $\alpha_4 = 27.64$ (ATF4), suggesting approximately equal contribution of these two TFs. However, besides these coefficients, the concentrations of ATF4 and pXBP1(S) themselves also have an important role in the contribution of the TFs to CHOP transcription. Because the amount of pXBP1(S) is much lower than ATF4 (Fig. S9), this concentration effect dominates when one considers the product terms $\alpha_3 X$, $\alpha_4 A$ and $\alpha_6 A_{6,h}$ and the pXBP1(s) contribution to CHOP transcription is small (Fig. 4B-D).

Choice of parameter ranges

During model calibration, we did not restrict the allowed parameter ranges taking full biophysical details into consideration because the units of the normalized intensities in our imaging data were arbitrary, precluding determination of the unit of concentrations. Rather, we required all parameters (besides s_s) to be positive and based our choices of parameter ranges on trial simulations with Trusina et al.'s previously published model parameterization (Trusina et al., 2008). The parameter ranges considered and the units of parameters are provided in Table S1. Note that the high values for some of the Michaelis-Menten constants are due to the high levels of unfolded proteins in our simulations (for which we have no measurements) and should be interpreted in a relative rather than absolute manner.

Covariance matrix of estimates

In order to study the uncertainty of the estimated parameter values, we utilize the Jacobian matrix ($\mathbf{J}$) to approximate the Hessian matrix $\mathbf{H}$:

$$\mathbf{H} = \mathbf{J}^T \mathbf{J}\, S(\theta)\,. \tag{28}$$

Here, the mean squared error $S(\theta)$ (i.e., the residual sum of squares divided by the number of degrees of freedom) is given by

$$S(\theta) = \frac{\mathbf{R}^T \mathbf{R}}{n_D - n_\theta}\,, \tag{29}$$

where R is the vector containing the residuals between model prediction and data, $n_D = 440$ denotes the number of data points and $n_\theta = 47$ denotes the number of free parameters in our

model with ATF6 branch. The squares of the standard errors of the estimates are the diagonal items in the co-variance matrix, expressed as the inverse matrix $\mathbf{H}^{-1}$. The standard errors of all parameters are presented in Fig. S3. We multiplied these values by 1.98 to obtain confidence intervals (CIs) (Raue et al., 2009) (shown together with the estimates in Table S1). Note that although the amount of experimental measurements is large compared to the number of parameters in our case, the CI for some parameters may be underestimated by the Hessian-based estimate.

Sensitivity analysis

We performed a sensitivity analysis to quantify the importance of the model parameters around our maximum likelihood estimate. Because CHOP is an important determinant in downstream cell fate and most of the signalling parameters are expected to indirectly affect the activity of CHOP especially around the peak, we focus on CHOP activity at 16 hours after treatment with 6μM of tunicamycin. Thus, we performed a local sensitivity analysis around the maximal likelihood estimate $\hat{\theta}$ for the model with ATF6.

In the sensitivity analysis, we omitted parameters that should have no impact on CHOP in conditions of 6μM tunicamycin treatment. Specifically, these are the effective tunicamycin concentrations of 2, 4, 12 μM, i.e. E_2, E_4 and E_{12}. Moreover, we omitted the three pairs of the scaling coefficients (e_{XBP1}, e_{ATF4}, and e_{BIP}) and offsets (s_{XBP1}, s_{ATF4}, and s_{BIP}) for the non-CHOP reporter cell lines. We varied each of the remaining parameters by both increasing and decreasing them by a small value (δ_θ) from its optimum. Subsequently, we quantified the sensitivity using the following equation:

$$\frac{\Delta C}{\Delta \theta_i} = \frac{C(\theta + \delta_\theta) - C(\theta - \delta_\theta)}{2\,\delta_\theta}\,, \tag{30}$$

where for different parameters, we used different δ_θ. For τ_2 and r_U, we chose a value just above the machine precision (3e-16); for other parameters, we set δ_θ to 1e-6$\times\hat{\theta}$, i.e., based on the maximum likelihood estimate. As the sensitivity can be negative or positive and the absolute value of sensitivities has a broad range, we plotted the log10 of the absolute values of the calculated sensitivities, with the colour indicating positive or negative sensitivity (Fig. S4). The most straightforward parameters having a positive impact on CHOP are the scaling parameters e_C and s_C, and parameters representing direct inputs like E_6 and e_s. As expected, parameters with a negative impact typically arise from those promoting degradation of CHOP, like r_C.

Supplemental References

Coleman, T. F., & Li, Y. (1996). An interior trust region approach for nonlinear minimization subject to bounds. *SIAM Journal on optimization*, *6*(2), 418–445.

Di, Z., Herpers, B., Fredriksson, L., Yan, K., van de Water, B., Verbeek, F. J., & Meerman, J. H. (2012). Automated analysis of nf-κb nuclear translocation kinetics in high-throughput screening. *PloS one*, *7*(12), e52337.

Diedrichs, D. R., Gomez, J. A., Huang, C.-S., Rutkowski, D. T., & Curtu, R. (2018). A data-entrained computational model for testing the regulatory logic of the vertebrate unfolded protein response. *Molecular biology of the cell*, mbc–E17.

Dowle, M., Srinivasan, A., Gorecki, J., Chirico, M., Stetsenko, P., Short, T., ... others (2018). Package 'data. table'.

Garcia-Alonso, L., Ibrahim, M. M., Turei, D., & Saez-Rodriguez, J. (2018). Benchmark and integration of resources for the estimation of human transcription factor activities. *bioRxiv*, 337915. doi: 10.1101/337915

García-Pérez, M. A., & Alcalá-Quintana, R. (2015a). Converging evidence that common timing processes underlie temporal-order and simultaneity judgments: A model-based analysis. *Attention, Perception, & Psychophysics*, *77*(5), 1750–1766.

García-Pérez, M. A., & Alcalá-Quintana, R. (2015b). Visual and auditory components in the perception of asynchronous audiovisual speech. *i-Perception*, *6*(6), 2041669515615735.

Hendriks, G., Atallah, M., Morolli, B., Calléja, F., Ras-Verloop, N., Huijskens, I., ... Vrieling, H. (2011). The toxtracker assay: novel gfp reporter systems that provide mechanistic insight into the genotoxic properties of chemicals. *Toxicological Sciences*, *125*(1), 285–298.

Hiemstra, S., Niemeijer, M., Koedoot, E., Wink, S., Klip, J. E., Vlasveld, M., ... Water, B. v. d. (2016). Comprehensive landscape of nrf2 and p53 pathway activation dynamics by oxidative stress and dna damage. *Chemical research in toxicology*, *30*(4), 923–933.

Love, M. I., Huber, W., & Anders, S. (2014). Moderated estimation of fold change and dispersion for rna-seq data with deseq2. *Genome biology*, *15*(12), 550.

Mav, D., Shah, R. R., Howard, B. E., Auerbach, S. S., Bushel, P. R., Collins, J. B., ... others (2018). A hybrid gene selection approach to create the s1500+ targeted gene sets for use in high-throughput transcriptomics. *PloS one*, *13*(2), e0191105.

McKay, M. D., Beckman, R. J., & Conover, W. J. (2000). A comparison of three methods for selecting values of input variables in the analysis of output from a computer code. *Technometrics*, *42*(1), 55–61.

Neuwirth, E. (2014). Rcolorbrewer: Colorbrewer palettes, r package version 1.1-2 http://cran. *R-project. org/package= RColorBrewer*.

Niemeijer, M., Hiemstra, S., Wink, S., den Hollander, W., ter Braak, B., & van de Water, B. (2018). Systems microscopy approaches in unraveling and predicting drug-induced liver injury (dili). In *Drug-induced liver toxicity* (pp. 611–625). Springer.

Poser, I., Sarov, M., Hutchins, J. R., Hériché, J.-K., Toyoda, Y., Pozniakovsky, A., ... others (2008). Bac transgeneomics: a high-throughput method for exploration of protein function in mammals. *Nature methods*, *5*(5), 409.

Raue, A., Kreutz, C., Maiwald, T., Bachmann, J., Schilling, M., Klingmüller, U., & Timmer, J. (2009). Structural and practical identifiability analysis of partially observed dynamical models by exploiting the profile likelihood. *Bioinformatics*, *25*(15), 1923–1929.

Raue, A., Schilling, M., Bachmann, J., Matteson, A., Schelke, M., Kaschek, D., ... others (2013). Lessons learned from quantitative dynamical modeling in systems biology. *PloS one*, *8*(9), e74335.

Rosenblatt, M., Timmer, J., & Kaschek, D. (2016). Customized steady-state constraints for parameter estimation in non-linear ordinary differential equation models. *Frontiers in cell and developmental biology*, *4*, 41.

Trusina, A., Papa, F. R., & Tang, C. (2008). Rationalizing translation attenuation in the network architecture of the unfolded protein response. *Proceedings of the National Academy of Sciences*, *105*(51), 20280–20285.

Wickham, H. (2010). ggplot2: elegant graphics for data analysis. *J Stat Softw*, *35*(1), 65–88.

Wickham, H. (2017). tidyr: Easily tidy data with'spread ()'and'gather ()'functions, 2017. *URL https://CRAN. R-project. org/package= tidyr. R package version 0.6*, *1*, 248.

Wickham, H., et al. (2011). The split-apply-combine strategy for data analysis. *Journal of Statistical Software*, *40*(1), 1–29.

Wink, S., Hiemstra, S., Herpers, B., & van de Water, B. (2017). High-content imaging-based bac-gfp toxicity pathway reporters to assess chemical adversity liabilities. *Archives of toxicology*, *91*(3), 1367–1383.

Wink, S., Hiemstra, S., Huppelschoten, S., Danen, E., Niemeijer, M., Hendriks, G., ... van de Water, B. (2014). Quantitative high content imaging of cellular adaptive stress response pathways in toxicity for chemical safety assessment. *Chemical research in toxicology*, *27*(3), 338–355.

Ye, J., Rawson, R. B., Komuro, R., Chen, X., Davé, U. P., Prywes, R., ... Goldstein, J. L. (2000). Er stress induces cleavage of membrane-bound ATF6 by the same proteases that process srebps. *Molecular cell*, *6*(6), 1355–1364.

Yeakley, J. M., Shepard, P. J., Goyena, D. E., VanSteenhouse, H. C., McComb, J. D., & Seligmann, B. E. (2017). A trichostatin a expression signature identified by tempo-seq targeted whole transcriptome profiling. *PLoS One*, *12*(5), e0178302.

Zhang, Z. (2016). Reshaping and aggregating data: an introduction to reshape package. *Annals of translational medicine*, *4*(4).
